# Cardiac Myosin‐Binding Protein C to Diagnose Acute Myocardial Infarction in the Pre‐Hospital Setting

**DOI:** 10.1161/JAHA.119.013152

**Published:** 2019-07-26

**Authors:** Thomas E. Kaier, Carsten Stengaard, Jack Marjot, Jacob Thorsted Sørensen, Bashir Alaour, Stavroula Stavropoulou‐Tatla, Christian Juhl Terkelsen, Luke Williams, Kristian Thygesen, Ekkehard Weber, Michael Marber, Hans Erik Bøtker

**Affiliations:** ^1^ King's College London BHF Centre The Rayne Institute St Thomas’ Hospital London United Kingdom; ^2^ Department of Cardiology Aarhus University Hospital Aarhus Denmark; ^3^ Institute of Physiological Chemistry Martin Luther University Halle‐Wittenberg Halle Germany

**Keywords:** cardiac myosin‐binding protein C, myocardial infarction, pre‐hospital triage, troponin T, Biomarkers, Coronary Artery Disease, Acute Coronary Syndromes

## Abstract

**Background:**

Early triage is essential to improve outcomes in patients with suspected acute myocardial infarction (AMI). This study investigated whether cMyC (cardiac myosin‐binding protein), a novel biomarker of myocardial necrosis, can aid early diagnosis of AMI and risk stratification.

**Methods and Results:**

cMyC and high‐sensitivity cardiac troponin T were retrospectively quantified in blood samples obtained by ambulance‐based paramedics in a prospective, diagnostic cohort study. Patients with ongoing or prolonged periods of chest discomfort, acute dyspnoea in the absence of known pulmonary disease, or clinical suspicion of AMI were recruited. Discrimination power was evaluated by calculating the area under the receiver operating characteristics curve; diagnostic performance was assessed at predefined thresholds. Diagnostic nomograms were derived and validated using bootstrap resampling in logistic regression models. Seven hundred seventy‐six patients with median age 68 [58;78] were recruited. AMI was the final adjudicated diagnosis in 22%. Median symptom to sampling time was 70 minutes. cMyC concentration in patients with AMI was significantly higher than with other diagnoses: 98 [43;855] versus 17 [9;42] ng/L. Discrimination power for AMI was better with cMyC than with high‐sensitivity cardiac troponin T (area under the curve, 0.839 versus 0.813; *P*=0.005). At a previously published rule‐out threshold (10 ng/L), cMyC reaches 100% sensitivity and negative predictive value in patients after 2 hours of symptoms. In logistic regression analysis, cMyC is superior to high‐sensitivity cardiac troponin T and was used to derive diagnostic and prognostic nomograms to evaluate risk of AMI and death.

**Conclusions:**

In patients undergoing blood draws very early after symptom onset, cMyC demonstrates improved diagnostic discrimination of AMI and could significantly improve the early triage of patients with suspected AMI.

**Clinical Perspective**

**What Is New?**

- In an observational, prospective diagnostic cohort study that included 776 individuals presenting with chest pain and suspected acute myocardial infarction (AMI), cMyC (cardiac myosin‐binding protein C) concentrations in blood draws obtained in the ambulance were significantly higher in patients with AMI than with other diagnoses.
- Discrimination power was significantly better for cMyC than for high‐sensitivity cardiac troponin T, and cMyC at the previously published threshold of 10 ng/L for rule‐out of AMI reached 100% sensitivity and negative predictive value in patients with only 2 hours of symptoms.

**What Are the Clinical Implications?**

- cMyC could significantly improve the early triage of patients with suspected AMI.
- We have developed a diagnostic nomogram, translating the combination of clinical risk factors and cMyC concentration into a personalized probability of AMI.

Rapid triage to the appropriate treatment is the cornerstone of improving outcome for patients presenting with suspected acute myocardial infarction (AMI).^1^, ^2^, ^3^ Prehospital and early hospital triage is, however, fraught with difficulties: The former relies heavily on the recording of ECGs and point‐of‐care measurement of biomarkers on devices that lack either cardiac specificity or the sensitivity of laboratory platforms. Early hospital triage is restrained by the biology of cardiac troponin (cTn), reflected in guidelines enabling direct rule‐out of myocardial infarction only from at least 3 hours after symptom onset.^1^ To streamline acute cardiac care, physicians at Aarhus University Hospital (Denmark) evaluate over 6000 prehospital ECGs per year, transmitted from paramedics in the field. This system allows the team in the regional tertiary care interventional center to select the cases for priority transfer, bypassing the nearest secondary care facility.^4^ However, ECG abnormalities identify only a minority of cases of AMI, do not allow risk stratification,^5^ and the interpretation is compounded by bundle branch block and other long‐standing abnormalities.^4^ A recent study investigating the precision with which emergency staff interpret ECGs (including ST‐elevation) has demonstrated a mean accuracy of 81% across all study groups (such as paramedics, residents, and cardiologists).^6^ For patients with high‐risk non–ST‐segment–elevation myocardial infarction (NSTEMI), the inherent diagnostic challenges lead to delayed appropriate treatment and may be associated with worse outcomes.^7^ In a recent study, the end‐point committee readjudicated 9% to 14% of NSTEMI patients as ST‐segment–elevation myocardial infarction (STEMI), challenging the perception that ECG‐based triage by a hospital physician is sufficient to identify all patients benefiting from urgent revascularization.^7^

We have previously studied the performance of cardiac troponin T (cTnT) and copeptin point‐of‐care testing (POCT) devices to aid triage in the prehospital setting. Although cardiac specific,^8^ the cTnT POCT assay has a lower limit of quantification (LoQ) of 50 ng/L, with a 99th centile, defined by laboratory platforms, of 14 ng/L. Copeptin, on the other hand, is released early after acute illness, but low specificity limits its use in guiding patients toward regional interventional cardiology centers.^9^

We previously described cMyC (cardiac myosin‐binding protein C)—a novel biomarker of myocardial necrosis and a more abundant analyte than cTn.^10^, ^11^ In smaller studies investigating patients early after chest pain onset or timed cardiac injury, cMyC rises more rapidly than cardiac troponin I^11^, ^12^—at equal, absolute tissue specificity. In a recently published study,^13^ cMyC demonstrated favorable classification into rule‐out and rule‐in categories when compared with high‐sensitivity cTn. Specifically, the reclassification improvement was more pronounced in patients presenting early after chest pain onset (≤3 hours). In combination, these features make cMyC an attractive biomarker for POCT. Recent correspondence demonstrated interest in the biomarker's discrimination power in very early presenters, irrespective of ECG findings.^14^, ^15^ Using conventional performance metrics as well as the development of diagnostic and prognostic nomograms, this study investigated whether cMyC—tested in a cohort of patients undergoing in‐ambulance blood draws—could aid the early diagnosis of AMI.

## Methods

### Study Design and Population

In an observational, prospective, quality‐control study, paramedics routinely performed point‐of‐care cTnT measurements in patients with suspected AMI.^16^ The point‐of‐care cTnT measurements were performed in 25 ambulances in the eastern part of the Central Denmark Region with a population of ≈600 000 inhabitants from May 26, 2010 to May 16, 2011. Each patient in whom the standard operating procedure instructed the recording of a prehospital ECG qualified for blood testing. The standard operating procedure criteria included ongoing or prolonged periods of chest discomfort within the past 12 hours, acute dyspnea in the absence of known pulmonary disease, or clinical suspicion of AMI. The study was reviewed by the Regional Ethical Committee and accepted as a quality‐control study. Oral informed consent for participation in the study was obtained in the ambulance. The study was approved by the Danish Data Protection Agency and the Danish National Board of Health. The methods used in this analysis are available from the corresponding author.

### Telemedicine Triage

The ECG was transmitted to the invasive cardiology center at Aarhus University Hospital, Denmark, and interpreted by the cardiologist on call. Subsequently, a telephone interview was conducted with the patient. Thereafter, a tentative cardiac or a noncardiac diagnosis was established and the patient underwent triage to either the percutaneous coronary intervention center or a local hospital for further assessment.^4^

Following point‐of‐care cTnT analysis, the paramedics saved the remaining blood sample obtained in the ambulance. For details on sample storage and analysis, as well as data sources, please see Data S1.

### Cardiac Biomarker Analysis

cMyC was measured using the previously established high‐sensitivity assay on the Erenna platform and was performed by Millipore Sigma (Hayward, CA).^17^ The assay has a lower limit of detection (LoD) of 0.4 ng/L and a lower limit of quantification (LoQ) of 1.2 ng/L with a ≤20% coefficient of variation at LoQ, and ≤10% coefficient of variation at the 99th centile. Assay precision is not affected by freeze/thaw cycles, and results are closely correlated across different matrices (serum, lithium heparin, and K2 EDTA).^17^ The estimated 99th percentile cut‐off point (upper reference limit) determined previously is 87 ng/L.^17^ The precision profile is displayed in Figure S1 and Table S1 and remains ≤10% above 4.6 ng/L. We have recently contracted a POCT diagnostics device manufacturer to migrate cMyC onto their platform. As demonstrated in Figure S2, our proposed threshold of 10 ng/L is attainable with a coefficient of variation ≤10% on a precommercial device.^13^

For high‐sensitivity cardiac troponin T (hs‐cTnT), samples were thawed and analyzed as 1 batch in a “thaw‐freeze” cycle at the central laboratory of Aarhus University Hospital, using the hs‐cTnT assay (Roche Diagnostics GmbH, Mannheim, Germany). The assay has an LoD of 5 ng/L, with a coefficient of variation ≤10% at 13 ng/L and the 99th centile at 14 ng/L.^18^ Roche Diagnostics has previously released a technical bulletin regarding a calibration issue affecting all lots used in this study and for routine hs‐cTnT measurements made during hospital admission.^19^, ^20^ The manufacturer recommended a method for recalculating the reported values using combined calibration information, reagent lot number information, and instrument details if the original signal data were not available.^21^ Where available, hs‐cTnT samples below the 99th centile were subsequently reanalyzed using reagent lots unaffected by the calibration issue to avoid ambiguities attributed to recalculation (n=287). A number of samples (n=202) have recalculated hs‐cTnT concentrations—most of which affect samples with hs‐cTnT values above the 99th centile. The hs‐cTnT recovery rate and the 99th centile comply with those found in the original studies.^18^, ^19^, ^21^

### Adjudicated Final Diagnosis

As previously described, all admissions were reviewed by an end‐point committee for adjudication of the final diagnosis.^16^ This was performed according to the universal definition of myocardial infarction.^22^ For the diagnosis of myocardial injury, the hs‐cTnT upper reference limit was used. hs‐cTnT values obtained from prehospital samples were not disclosed or used in clinical decision making. The end‐point committee had access to all patient file material, including the discharge file, with the diagnoses determined by the clinicians. AMI patients were classified as STEMI or NSTEMI; unstable angina was diagnosed in patients with a significant episode of chest pain thought to be of ischemic origin who did not fulfil AMI criteria.

The cardiologist on call recorded clinical and baseline data as well as the triage decision using a web‐based telemedicine database. Prehospital data were obtained from the Central Denmark Region's Prehospital Emergency Medical Services. Clinical details and baseline data were acquired from patient files in hard copies from the hospitals and from The National Patient Registry. Survival data were obtained from The Danish Civil Registration System. Baseline health information was obtained from The National Patient Registry. At 30 days, 2 independent adjudicators evaluated all prehospital, in‐hospital, and survival data. AMIs without cardiac death during 30‐day follow‐up were classified a nonfatal AMI.

### Diagnostic Proportions of hs‐cTnT and cMyC

Classification power of both biomarkers was assessed by calculating sensitivity, negative predictive value, specificity, and positive predictive value for each cut‐off threshold. The 99th centile of hs‐cTnT is 14 ng/L, and the currently available POCT platform (Roche Cobas h323 hand‐held instrument) can detect a laboratory‐equivalent value of 50 ng/L (POCT LoD, correct at date of submission)—approximately 3‐fold the LoQ or 10‐fold the LoD of the laboratory assay.^23^ The result is reported as “negative” <50 ng/L, “positive” at 50% to 100 ng/L, and quantitatively positive with a numerical value >100 ng/L.

In line with results from a first foray into detection of cMyC concentrations on a POCT platform (see Data S1 and Figure S1), 10 ng/L (the previously published threshold for rule‐out of AMI^13^) seems feasible. We used 1000 bootstrap replicates to determine the classification power of each biomarker with 95% CIs. Net reclassification improvement and integrated discrimination improvement were calculated in line with Pencina's recommendations.^24^ A positive net reclassification improvement indicates an improvement of classification from the initial model: Categorical net reclassification improvement equal to x% means that compared with individuals without outcome, individuals with outcome were almost x% more likely to move up a category than down. Integrated discrimination improvement equal to x% means that the difference in average predicted risks between the individuals with and without the outcome increased by x% in the updated model.

### Statistical Analysis

All data are expressed as medians [first quartile; third quartile] or means (SD) for continuous variables (compared with a *t* test or ANOVA for continuous normal distributed variables and Kruskal–Wallis test if continuous non‐normally distributed); categorical variables are expressed as absolute and relative frequencies (compared with Pearson chi‐square). Hypothesis testing was 2‐tailed, and *P*<0.05 was considered statistically significant. Where bootstrap techniques were used, the calculations were performed using 1000 stratified replicates.

Diagnostic accuracy was quantified by the area under the receiver operating curve (AUC [95% CI]) against adjudicated AMI. Bootstrapping was used to calculate CIs, compare the AUC between biomarkers, and calculate the classification function. Youden's index was calculated to quote the concentration at which the sum of sensitivity and specificity is maximized.^25^ Logistic regression was used to combine cMyC with hs‐cTnT values for the assessment of an incremental value using the 2 biomarker concentrations at presentation. Correlation was assessed with Spearman's rho (r_s_) and adjusted *R* ^2^ by fitting a linear regression model.

#### Regression models

Several regression models incorporating available biomarker concentrations (hs‐cTnT and cMyC) and clinical variables (history of diabetes mellitus, hyperlipidemia, hypertension, smoking, and previous myocardial infarction; baseline variables sex, age, and creatinine) were evaluated—(1) logistic regression models for the adjudicated diagnosis of AMI upon index presentation and (2) Cox proportional hazard models to predict probability of (a) death and (b) nonfatal AMI or death during follow‐up.

We used restricted cubic splines to model the distribution of cMyC, given that the assay was able to detect a cMyC concentration in every enrolled participant tested and thus no individual was below the LoD (0.4 ng/L). For hs‐cTnT, we modeled the distribution using linear splines—all concentrations below LoD (5 ng/L) were assigned the value 4.99 ng/L, and the knot locations were assigned at quantiles 5%, 25%, 50%, and 75% above the LoD.

A short model for the probabilistic assessment of AMI likelihood was derived using a pragmatic approach informed by fast backward variable selection. To assess probability of AMI, this resulted in the inclusion of the following factors for the derivation of a nomogram displayed in an abbreviated model suitable to the development of a nomogram: cMyC, sex, hyperlipidemia, and smoking history. Log likelihoods were used to quantify and compare the predictive information contained in each subset of predictors.

#### Prognostic models

Follow‐up was carried out for up to 2 years after enrollment to the study (recruitment period, May 26, 2010 to May 16, 2011). Cox regression models to predict probability of (1) death and (2) nonfatal AMI or death during follow‐up were derived using fast backward variable selection from a model including all baseline variables. To assess probability of death during follow‐up, this resulted in the inclusion of the following factors for the derivation of a nomogram: cMyC, creatinine, age, and previous history of myocardial infarction. The Cox models were tested for violation of the proportional hazards assumption by calculating correlation coefficients between transformed survival time (rank) and the scaled Schoenfeld residuals and testing the former with chi‐square comparisons. All available variables were tested in a univariate regression model; significant variables (predefined as Wald test *P*<0.1) were selected for the final Cox multivariate regression model. The biomarkers were entered log‐transformed.

All statistical analyses were performed using R software (version 3.3.0 GUI 1.68; The R Foundation for Statistical Computing, Vienna, Austria), including packages ggplot2, RMarkdown, the tidyverse, survival, survminer, and pROC.

## Results

### Baseline Characteristics

Samples from a total of 776 patients were available for retrospective analysis. Median age was 68 years [58; 78], 303 patients (39%) were women, and 232 (30%) had a previous history of myocardial infarction (Table 1 and Table S2). AMI was the adjudicated diagnosis in 173 patients (22%): 66 patients (9%) had a final diagnosis of STEMI and 107 (14%) NSTEMI. Median time since onset of chest pain was 70 minutes [35; 173]. In 99% of cases, a telephone consultation was undertaken. There was considerable discrepancy between telemedicine triage and final diagnosis: 107 patients (14%) presented with bundle branch block on ECG; only 59% of patients with a final adjudicated diagnosis of STEMI had clear ST‐elevation identified during telemedicine assessment. Sensitivity for NSTEMI during telemedicine assessment was 33%.

**Table 1:** Baseline Characteristics Stratified by AMI Diagnosis

	No AMI (N=603)	AMI (N=173)	*P* Value*	N
Sex: male	344 (57%)	129 (75%)	<0.001	776
Age, y	68 [56; 78]	70 [63; 79]	0.016	776
Hypertension	337 (56%)	102 (59%)	0.528	776
Hyperlipidemia	480 (80%)	142 (82%)	0.540	776
Diabetes mellitus	124 (21%)	23 (13%)	0.041	776
Current smoking	165 (31%)	65 (46%)	0.001	674
Past smoking	167 (31%)	50 (35%)	0.445	674
Previous MI	174 (29%)	58 (34%)	0.276	776
Previous percutaneous intervention	151 (25%)	49 (28%)	0.440	776
Systolic blood pressure, mm Hg	146 [130; 165]	149 [129; 170]	0.531	764
Diastolic blood pressure, mm Hg	87 [75; 98]	89 [73; 105]	0.154	764
Heart rate, bpm	84 [70; 100]	85 [70; 100]	0.790	765
eGFR, mL/min/1.73 m^2^ *	72 [56; 87]	68 [58; 83]	0.126	605
Time since chest pain onset, min	66 [35; 179]	72 [35; 150]	0.872	726

AMI indicates acute myocardial infarction; eGFR, estimated glomerular filtration rate; MDRD, Modification of Diet in Renal Disease; MI, myocardial infarction.

*P values for comparison AMI group vs all other diagnoses; data are expressed as medians [first quartile, third quartile], for categorical variables as numbers (percentages); eGFR in mL/min per 1.73 m^2^, estimated using the MDRD formula; *P* value for comparison AMI vs non‐AMI.

### Distribution of Biomarker Concentrations

**Figure 1:**
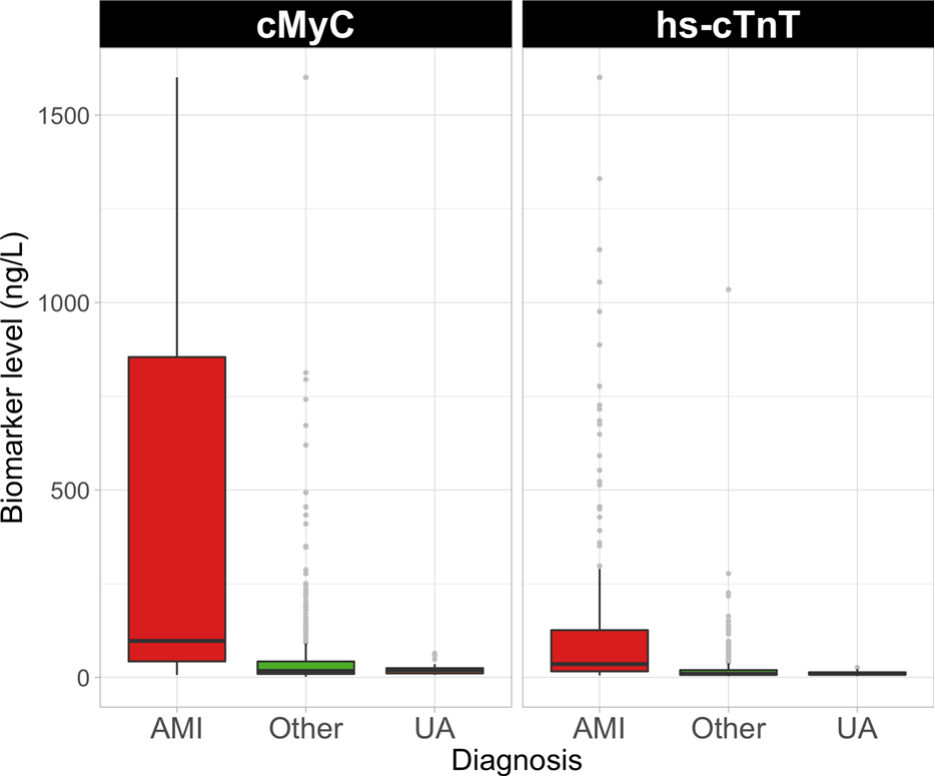
Distribution of cMyC and hs‐cTnT concentrations in samples obtained in the ambulance, based on adjudicated final diagnosis. Boxes represent interquartile ranges; whiskers extend to 1.5×IQR from the hinges; light gray bullets are outliers. AMI indicates acute myocardial infarction; cMyC, cardiac myosin‐binding protein C; hs‐cTnT, high‐sensitivity cardiac troponin T; IQR, interquartile range; UA, unstable angina.

All blood samples were obtained in the ambulance, but measured in a laboratory for hs‐cTnT and cMyC. In‐ambulance concentrations of cMyC were significantly higher in patients with AMI (median, 98 ng/L [43; 855]) than in patients with other diagnoses (17 ng/L [9; 42]; *P*<0.001). Median concentrations of cMyC were 88 ng/L [42; 253] for NSTEMI, 306 ng/L [49; 1706] for STEMI, and 19 ng/L [11; 25] for unstable angina. The corresponding concentrations for hs‐cTnT were 33 ng/L [18; 72], 58 ng/L [15; 295], and 9 ng/L [7; 14], respectively (see Figure 1; Table S3). In this cohort, there was a slight sex difference in cMyC concentration in patients without AMI: female 15 ng/L [8; 38] versus male 18 ng/L [10; 44]; *P*=0.023; the difference does not reach significance in patients with AMI (female 121 ng/L [67; 1120] versus male 91 ng/L [38; 739]; *P*=0.235). Correlation between hs‐cTnT and cMyC is shown in Figure S3 and Table S4.

**Figure 2:**
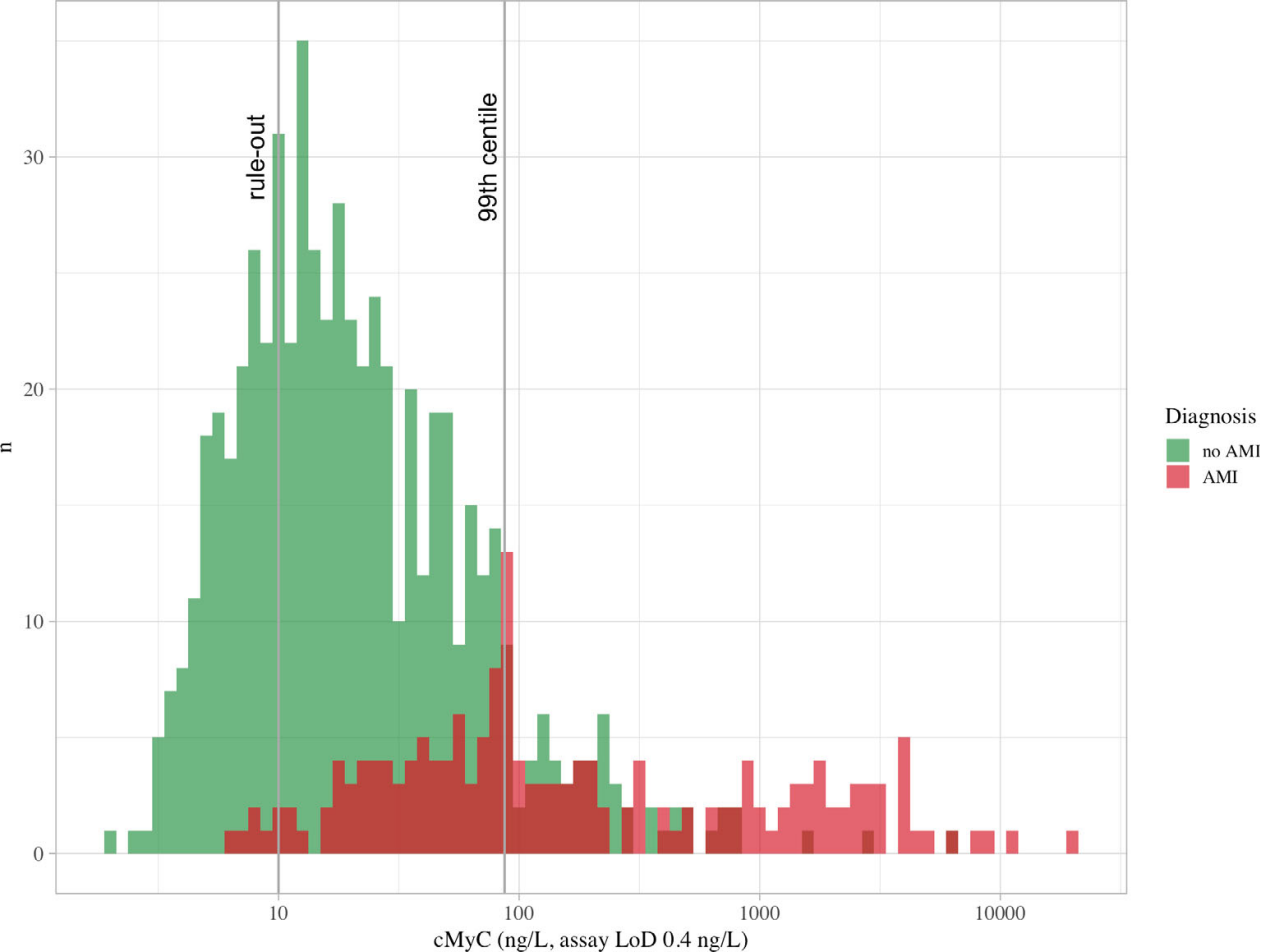
Distribution of patients stratified by adjudicated diagnosis of AMI based on the prehospital cMyC concentration. *x*‐axis log_10_‐transformed. AMI indicates acute myocardial infarction; cMyC, cardiac myosin‐binding protein C; LoD, lower limit of detection.

An overview of the distribution of cMyC is shown in Figure 2 (Figure S4 for hs‐cTnT). Overall, when comparing blood concentrations of biomarkers to assay specifics (LoQ, LoD), cMyC concentrations were higher than those of hs‐cTnT in all diagnostic categories.

### Discrimination Power for Use of Biomarkers Alone

**Figure 3:**
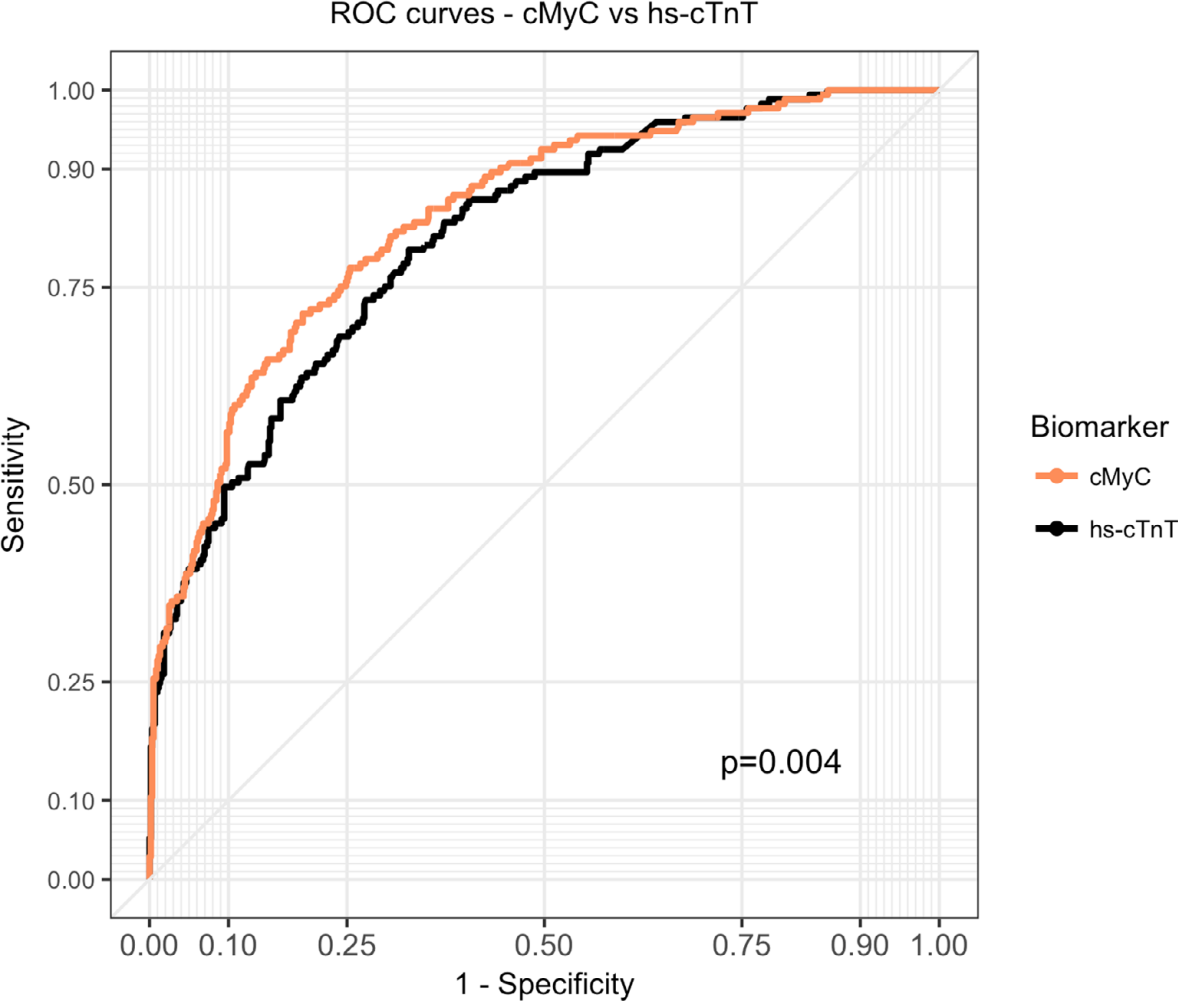
Receiver‐operating characteristics (ROC) curves for cMyC (ambulance) and hs‐cTnT (ambulance) for the diagnosis of acute myocardial infarction. The AUC for cMyC was 0.839 (95% CI, 0.804–0.87), for hs‐cTnT 0.813 (0.777–0.847). Youden's index for cMyC in this cohort is 50 ng/L. AUC indicates area under the curve; cMyC, cardiac myosin‐binding protein C; hs‐cTnT, high‐sensitivity cardiac troponin T.

In blood draws performed in the ambulance, the discrimination power against ultimate diagnosis (AMI) as quantified by the AUC was higher for cMyC than for hs‐cTnT: 0.839 (95% CI, 0.803–0.871) versus 0.813 (0.777–0.847; *P*=0.005 for direct comparison; Figure 3; Table 2). The discrimination power of cMyC for the individual diagnoses was: AUC 0.816 (0.761–0.866) for STEMI, AUC 0.787 (0.741–0.829) for NSTEMI, and AUC 0.599 (0.531–0.67) for unstable angina; Youden's index was calculated at 50 ng/L.

**Table 2:** Area Under the Receiver Operating Characteristics Curve for cMyC and hs‐cTnT

Outcome	AUC	95% CI	AUC	95% CI	Cases	Controls	*P* Value
Biomarker	cMyC		hs‐cTnT				
AMI	0.839	0.805 – 0.873	0.813	0.777 – 0.847	173	603	0.005
STEMI	0.816	0.759 – 0.865	0.766	0.695 – 0.831	66	710	<0.001
NSTEMI	0.787	0.742 – 0.828	0.781	0.737 – 0.821	107	669	0.599
UA	0.599	0.524 – 0.670	0.608	0.531 – 0.690	27	749	0.715
Biomarker	cMyC+hs‐cTnT		hs‐cTnT				
AMI	0.822	0.791 – 0.856	0.813	0.775 – 0.847	173	603	<0.001
STEMI	0.780	0.716 – 0.836	0.766	0.699 – 0.834	66	710	<0.001
NSTEMI	0.786	0.744 – 0.830	0.781	0.738 – 0.823	107	669	0.041
UA	0.613	0.535 – 0.695	0.608	0.530 – 0.693	27	749	0.377

AMI indicates acute myocardial infarction; AUC, area under the curve; cMyC, cardiac myosin‐binding protein C; hs‐cTnT, high‐sensitivity cardiac troponin T; NSTEMI, non–ST‐segment–elevation myocardial infarction; STEMI, ST‐segment–elevation myocardial infarction; UA, unstable angina.

The discrimination power for hs‐cTnT for the individual diagnoses was: AUC 0.766 (0.701–0.828; *P*<0.001 for direct comparison to cMyC) for STEMI, AUC 0.781 (0.737–0.820; *P*=0.595) for NSTEMI, and AUC 0.608 (0.529–0.692; *P*=0.711) for unstable angina (Figures S5 and S6 for receiver operating characteristic curves). A stratified analysis based on time since symptom onset is shown in Table S5.

The combination of both markers (cMyC and hs‐cTnT) provided incremental value for STEMI (AUC 0.780; 0.719–0.84; *P*<0.001 for direct comparison) and NSTEMI (0.786; 0.745–0.824; *P*=0.037) compared with using hs‐cTnT alone.

### Logistic Regression Models for AMI Diagnosis

A model using all available biomarkers achieved a moderate model fit (*R* ^2^ 0.483), but a higher C index (C 0.875) and log likelihood ratio (LR; χ^2^ 291.5) than using the respective biomarkers alone. Figure S7 depicts the odds ratio for AMI diagnosis at presentation stratified by sex, creatinine, and cMyC concentrations, while holding other variables stable (Table S6 for regression model, calibration plot Figure S8). Models using only 1 (cardiac) biomarker yield lower discrimination indices than the model using both biomarkers (cMyC –*R* ^2^ 0.467, C 0.868, LR χ^2^ 282.4; hs‐cTnT –*R* ^2^ 0.431, C 0.853, LR χ^2^ 256.9).

However, based on the comparison of log likelihoods, the model including cMyC explains a greater proportion of the complete model than hs‐cTnT (difference in LR, χ^2^=25.5) and thus carries greater diagnostic information (Table S7).

### Development of a Nomogram for Prediction of AMI

**Figure 4:**
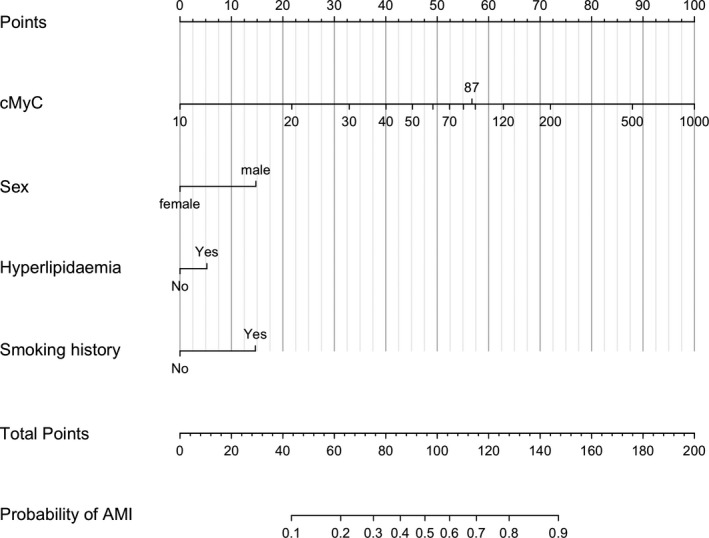
Nomogram for the use of cMyC concentration, sex, hyperlipidemia, and smoking history to predict probability of AMI. For example, a patient with cMyC concentration <10 ng/L would score 0 points or 100 points at a concentration of 1000 ng/L. Presence of hyperlipidemia would add 5 points; all points are added for the total score, which can then provide a probability of AMI. AMI indicates acute myocardial infarction; cMyC, cardiac myosin‐binding protein C.

Four variables remained in the final, short model used for the development of a nomogram (Figure 4): cMyC, sex, hyperlipidemia, and smoking history. Model statistics are displayed in Table S8 (Table S9 for validation) and, in short, achieved the following indices: *R* ^2^ 0.416, C 0.852.

### Diagnostic Proportions of cMyC and hs‐cTnT

Performance characteristics for cMyC at previously published thresholds^13^ for rule‐out (10 ng/L) and rule‐in (120 ng/L) of myocardial infarction, as well as the 99th centile (87 ng/L), are displayed in Table 3, stratified by symptom time (<60, 60–120, and >120 minutes of chest pain); for all patients across the cohort, see Table S10. The performance characteristics of hs‐cTnT were previously reported^16^ and are listed at 99th centile (14 ng/L), LoD of the high‐sensitivity assay (5 ng/L), and POCT device (50 ng/L) and rule‐in for myocardial infarction as per European Society of Cardiology guideline (52 ng/L) in Tables S11 and S12.^1^ In short, the rule‐out threshold for cMyC (10 ng/L) achieves sensitivity and negative predictive value of 100% after 2 hours of chest pain. For all patients, specificity at the 99th centile (87 ng/L) was 90.2% (87.6–92.6) and positive predictive value 61.4% (54–69.6); at the rule‐in threshold (120 ng/L), specificity was 92.2% (90–94.3) and positive predictive value 62.7% (54.6–71.3). A reclassification analysis is presented in Table S13, indicating an improvement in classification (based on net reclassification improvement +0.1067 and integrated discrimination improvement +0.032) by using cMyC instead of hs‐cTnT as the triage biomarker.

**Table 3:** Discriminatory Power of cMyC at Different Thresholds

[cMyC]			
	10 ng/L	87 ng/L	120 ng/L
Patients with chest pain for <60 min (n=321)			
Sensitivity	94.3% (87%–98.6%)	40.7% (29.1%–52.3%)	33.3% (22.2%–44.7%)
Specificity	32.1% (26.8%–37.9%)	90.3% (86.6%–93.8%)	92.3% (88.8%–95.3%)
NPV	95.5% (90.7%–98.9%)	85.6% (81.1%–89.6%)	84.4% (79.7%–88.5%)
PPV	26.3% (21%–32%)	52.1% (37.8%–65.4%)	52.5% (37.5%–67.5%)
Patients with chest pain for 60 to 120 min (n=156)			
Sensitivity	98.1% (93.9%–100%)	54.7% (41.5%–68.5%)	46.5% (33.3%–60.8%)
Specificity	22.8% (15%–31.3%)	92.7% (86.9%–96.9%)	92.7% (86.9%–96.9%)
NPV	96.4% (87.1%–100%)	80.9% (73.6%–87.2%)	78.3% (70.6%–84.9%)
PPV	38% (30.3%–46.3%)	78% (63.9%–89.8%)	75% (58.6%–88.5%)
Patients with chest pain for ≥120 min (n=249)			
Sensitivity	100% (100%–100%)	73.5% (61.8%–84.6%)	61.2% (48.3%–75%)
Specificity	29.9% (23.8%–36.6%)	88.9% (84%–93%)	91.5% (87.3%–95.1%)
NPV	100% (100%–100%)	92.7% (88.6%–96.2%)	90% (85.5%–93.8%)
PPV	27.6% (21.1%–33.7%)	63.8% (51.2%–75.5%)	65.8% (52.2%–78.4%)

cMyC indicates cardiac myosin‐binding protein C; NPV, negative predictive value; PPV, positive predictive value.

### Prediction of Death and First of Nonfatal Myocardial Infarction/Death During Follow‐up

Patients were followed for up to 2 years after the index presentation: Of the 173 patients with AMI, 28 (16%) died during follow‐up; of the patients without AMI, 60 (10%) died. An abbreviated model to predict death during follow‐up used the following factors for the derivation of a nomogram: cMyC, creatinine, age, and previous myocardial infarction. Model statistics are displayed in Table S14 and, in short, achieved the following indices: *R* ^2^ 0.179, C 0.798. For the model predicting nonfatal AMI or death during follow‐up, factors cMyC and history of diabetes mellitus were included and achieved *R* ^2^ 0.317, C 0.828 (Figures S9 through S11; Table S15 for hazard ratios; Figure S12 for event curves).

## Discussion

cMyC is a myocardial protein that is released into the circulation after injury in a similar manner to the cTn. A previous publication has suggested that the concentration of cMyC rises more rapidly than cTn based on an analysis of 26 patients with AMI, who presented to the hospital within 180 minutes of symptom onset.^12^ This finding is in keeping with an in vitro analysis of the human heart that shows that cMyC is many times more abundant than cTn.^10^ A recent investigation has further shown superiority in early triage of >1900 patients presenting with chest pain and suspected AMI—particularly in subjects presenting early after symptom onset.^13^ The median chest pain duration before first blood draw is typically 3 to 5 hours in large cohort studies undertaken in the secondary‐care setting.^26^, ^27^ In contrast, we studied patients with a median time of just 70 minutes between symptom onset and blood draw in the ambulance—a population enriched for AMI, attributed to the circumstance of recruitment. The current study indicates superiority of the novel biomarker in the rule‐out and diagnosis of AMI very early, based on an analysis of receiver operator characteristics, logistic regression modeling, and log LRs. Our direct observations and hypothetical models suggest that cMyC may have distinct advantages as a point‐of‐care biomarker for AMI. This advantage of cMyC is evident despite the use of hs‐cTnT in the final adjudication of AMI.

A biomarker result obtained in the prehospital setting or at first arrival to the hospital could be interpreted with simple decision aids, such as a nomogram that translates the biomarker value plus cardiovascular risk factors into a probability of AMI. Alternatively, established risk stratification tools, such as the Global Registry of Acute Coronary Events (GRACE) risk score,^28^ can be used to identify patients with NSTEMI who benefit from early revascularization—but the calculator requires an abnormal biomarker result obtained swiftly to classify high‐risk acute coronary syndrome. In the data presented, even the diagnosis of STEMI was far from certain—thus it is intriguing that the AUC for the diagnosis of STEMI is higher for cMyC than it is for hs‐cTnT, while not statistically different for NSTEMI in this cohort. Notably, cMyC provided incremental value to hs‐cTnT measurement alone in all AMI categories. Patients identified earlier as high risk could be transferred to the nearest percutaneous coronary intervention–capable facility, whereas low‐risk patients—with a low likelihood of AMI—are admitted locally.

Currently, the way prehospital triage is performed is resource intensive and yields imperfect results—ECGs have particularly low sensitivity in the context of (more common^5^) NSTEMI presentations, and the best commercially available POCT platforms for cTn have limits of quantification that are well above the population 99th centile defined using a laboratory assay. This limitation is part technology, part relative scarcity of the analyte—while a recent publication demonstrates a possible breakthrough with a portable high‐sensitivity cardiac troponin I assay, regulatory approval and full disclosure on true assay performance are eagerly awaited.^29^ Furthermore, the latest European Society of Cardiology guidelines^1^ specifically warn against the use of high‐sensitivity troponin assays in early presenters (<3 hours of chest pain). A protein which is much more abundant than cTn following myocardial injury would allow careful titration to individual requirements: Whether the goalpost is maximum specificity/positive predictive value, or maximum sensitivity/negative predictive value, such as in rapid rule‐in and rule‐out pathways—the greater the “detectable” spectrum of concentrations of an equally cardiac‐specific marker, the greater the possibility to choose cutoffs to achieve local objectives. Our analysis has demonstrated that a cMyC concentration <10 ng/L might be sufficient after 2 hours of symptoms to reliably rule‐out AMI; notably, this concentration is approximately 25‐fold the LoD of the current assay, which would allow for significant signal loss in the migration to POCT and still provide a useful tool for risk stratification. Furthermore, previously published rule‐in thresholds^13^ (120 ng/L) demonstrate a comparably high specificity (>90%) irrespective of symptom onset.

This study has several limitations: (1) cMyC is currently only available on a high‐sensitivity research platform, and the migration onto POCT has not been completed. (2) Any cutoffs investigated are subject to cohort‐specific calibration—hence, the current analysis utilizes additional, agnostic approaches such as the application and comparison of logistic regression models, which are not dependent on assay‐specific cutoffs. To allow a more clinically relevant interpretation, the information provided has been translated into diagnostic nomograms—to calculate probabilities of AMI or death based on an individual's cMyC concentrations plus clinical variables. The ability to detect lower volumes of myocardial injury earlier might be of particular use in a cohort such as the one studied, where the median time since onset of chest pain is substantially lower than in other, diagnostic chest pain studies, and rule‐in of high‐risk cases is of much greater importance to both the clinician and the patient. The clinical utility of the nomograms, however, is uncertain until validated in external cohorts. Furthermore, implementation would require a sensitive cMyC assay on a point‐of‐care platform; such a platform is not currently available. (3) As in most studies of this type, there is an inherent bias against the new biomarker given that high‐sensitivity troponin T was measured during the in‐hospital course and used in the clinical adjudication of AMI.

In summary, we have demonstrated that: (1) cMyC achieves improved diagnostic discrimination at earlier time points compared with hs‐cTnT; (2) the addition of cMyC to hs‐cTnT would provide additional diagnostic information; and (3) cMyC achieves high sensitivity and negative predictive value at 10 ng/L, a relatively high concentration that may be measurable at point of care.

## Sources of Funding

Dr Stengaard has received lecture fees and research grants from Roche Diagnostics (Basel, Switzerland), Thermo Fisher Scientific (Waltham, MA), and The Medicines Company (Parsippany, NJ). Dr Sørensen has received research grants from Falck Emergency Medical Services (Copenhagen, Denmark) and lecture fees from Roche Diagnostics. Dr Bøtker has received grants from the Danish Research Council (Copenhagen, Denmark). This work was further supported by grants from the Medical Research Council (London, UK; G1000737), Guy's and St Thomas’ Charity (London, UK; R060701, R100404), British Heart Foundation (Birmingham, London; TG/15/1/31518, FS/15/13/31320), and the UK Department of Health through the National Institute for Health Research Biomedical Research Centre award to Guy's & St Thomas’ National Health Service Foundation Trust.

## Disclosures

Millipore Sigma was contracted to undertake the analyses of cMyC on a fee‐for‐service basis and holds no commercial interest. Dr Marber is named as an inventor on a patent held by King's College London for the detection of cardiac myosin‐binding protein C as a biomarker of myocardial injury. The remaining authors have no disclosures to report.

## Supporting information


**Data S1.** Supplemental Methods.
**Table S1.** cMyC Precision Profile
**Table S2.** Baseline Characteristics Stratified by Final Diagnosis
**Table S3.** Distribution of Biomarker Concentration by Final Adjudicated Diagnostic Category
**Table S4.** Correlations Between cMyC and hs‐cTnT Concentrations by Diagnostic Group
**Table S5.** AUC Values for cMyC Versus hs‐cTnT Stratified by Time Since Symptom Onset: for Early (≤60 min), Intermediate (60–120 min), Late (≥120 min) Presenters
**Table S6.** Logistic Regression Model Statistics for Derivation of Figure S7
**Table S7.** Logistic Regression Models Incorporating All Variables, or cMyC and hs‐cTnT Alone
**Table S8.** Logistic Regression Model Statistics for cMyC
**Table S9.** Validation of Short cMyC Model Used for Nomogram Derivation
**Table S10.** Discriminatory Power of cMyC at Different Thresholds
**Table S11.** Discriminatory Power of hs‐cTnT at Different Thresholds—Stratified by Time Since Chest Pain Onset
**Table S12.** Discriminatory Power of hs‐cTnT at Different Thresholds—For All Patients
**Table S13.** Reclassification Analysis for cMyC Versus hs‐cTnT
**Table S14.** Prediction of Death and First Non‐Fatal MI/Death During Follow‐up
**Table S15.** Cox Regression Model for Outcome Death
**Figure S1.** cMyC assay precision profile.
**Figure S2.** Signal obtained for AgC (235‐3H8) against MgC (259‐1A4) for varying concentrations of C0C2 analyte.
**Figure S3.** Scatter plots outlining correlation between cMyC and hs‐cTnT concentrations (ng/L both) in samples obtained in the ambulance for each diagnostic group.
**Figure S4.** Histogram for hs‐cTnT concentrations from prehospital samples, stratified by diagnosis of AMI; *x*‐axis log_10_‐transformed.
**Figure S5.** Receiver operating characteristics (ROC) curves for cMyC (ambulance) and hs‐cTnT (ambulance) for the diagnosis of NSTEMI
**Figure S6.** Receiver operating characteristics (ROC) curves for cMyC (ambulance) and hs‐cTnT (ambulance) for the diagnosis of STEMI.
**Figure S7.** Odds ratio for AMI diagnosis at presentation based on [cMyC] and stratified by [creatinine]; facetted by sex (horizontal), and history of diabetes mellitus (vertical); other variables held stable.
**Figure S8.** Calibration plot for complete model, validated using 150 bootstrap repetitions.
**Figure S9.** Nomogram for the use of cMyC concentration, creatinine concentration, age, and history of previous myocardial infarction to predict probability of death during follow‐up.
**Figure S10.** Nomogram for the use of cMyC concentration and history of diabetes mellitus to predict probability of non‐fatal MI or death during follow‐up.
**Figure S11.** Facet plots describing effect of increasing cMyC concentration and previous myocardial infarction on the probability of death during follow‐up.
**Figure S12.** Cumulative event (mortality) curves for all patients over a 2‐year follow‐up for cMyC from samples obtained in the ambulance.
