# Supplementary material for: Cardiac Myosin‐Binding Protein C to Diagnose Acute Myocardial Infarction in the Pre‐Hospital Setting
Source: J Am Heart Assoc. 2019 Jul 26;8(15):e013152. doi: 10.1161/JAHA.119.013152 (PMC6761674; doi:10.1161/JAHA.119.013152)
Supplement: Supplementary file 1 — Data S1. Supplemental Methods. Table S1. cMyC Precision Profile Table S2. Baseline Characteristics Stratified by Final Diagnosis Table S3. Distribution of Biomarker Concentration by Final Adjudicated Diagnostic Category Table S4. Correlations Between cMyC and hs‐cTnT Concentrations by Diagnostic Group Table S5. AUC Values for cMyC Versus hs‐cTnT Stratified by Time Since Symptom Onset: for Early (≤60 min), Intermediate (60–120 min), Late (≥120 min) Presenters Table S6. Logistic Regression Model Statistics for Derivation of Figure S7 Table S7. Logistic Regression Models Incorporating All Variables, or cMyC and hs‐cTnT Alone Table S8. Logistic Regression Model Statistics for cMyC Table S9. Validation of Short cMyC Model Used for Nomogram Derivation Table S10. Discriminatory Power of cMyC at Different Thresholds Table S11. Discriminatory Power of hs‐cTnT at Different Thresholds—Stratified by Time Since Chest Pain Onset Table S12. Discriminatory Power of hs‐cTnT at Different Thresholds—For All Patients Table S13. Reclassification Analysis for cMyC Versus hs‐cTnT Table S14. Prediction of Death and First Non‐Fatal MI/Death During Follow‐up Table S15. Cox Regression Model for Outcome Death Figure S1. cMyC assay precision profile. Figure S2. Signal obtained for AgC (235‐3H8) against MgC (259‐1A4) for varying concentrations of C0C2 analyte. Figure S3. Scatter plots outlining correlation between cMyC and hs‐cTnT concentrations (ng/L both) in samples obtained in the ambulance for each diagnostic group. Figure S4. Histogram for hs‐cTnT concentrations from prehospital samples, stratified by diagnosis of AMI; x‐axis log10‐transformed. Figure S5. Receiver operating characteristics (ROC) curves for cMyC (ambulance) and hs‐cTnT (ambulance) for the diagnosis of NSTEMI Figure S6. Receiver operating characteristics (ROC) curves for cMyC (ambulance) and hs‐cTnT (ambulance) for the diagnosis of STEMI. Figure S7. Odds ratio for AMI diagnosis at presentation based on [cMyC] and stratified b [file JAH3-8-e013152-s001.pdf]

SUPPLEMENTAL MATERIAL

Data S1.

Supplemental Methods

Sample storage and analysis

The sample was initially stored at 4˚C in the ambulance and later stored in refrigerators at Aarhus University Hospital. Laboratory personnel collected the blood samples from the refrigerators periodically at intervals of a maximum of 12h, centrifuged the samples, and stored the plasma at -80˚C. The Central Denmark Region Committees on Biomedical Research Ethics reviewed the protocol and approved the study as a biological registry study. Handling of patient data and storage of the blood samples were reported to the Danish Data Protection agency. Clinical data were reviewed with permission from the Danish National Board of Health. Both high-sensitivity assays, hs-cTnT and cMyC, were performed using laboratory analysers on stored plasma samples. The POCT cTn readings are not included in our analysis.

Data sources

The cardiologist on call used a web-based telemedicine database to record clinical, baseline demographic and timing data, as well as the tentative diagnosis, ECG changes and triage decision. Timings were obtained from the Central Denmark Region’s Prehospital Emergency Medical Services. Clinical details and demographic data were acquired using hard copies of patient files and from the National Patient Registry. Symptom duration was calculated using the difference between recorded symptom onset to prehospital blood sampling time point. Follow-up data to assess survival was obtained from The Danish Civil Registration System. electrocardiogram recorded.

Supplemental Results

STARD checklist

Section & Topic	No	Item	Reported on page #
TITLE OR ABSTRACT			
	1	Identification as a study of diagnostic accuracy using at least one measure of accuracy<br>(such as sensitivity, specificity, predictive values, or AUC)	3
ABSTRACT			
	2	Structured summary of study design, methods, results, and conclusions<br>(for specific guidance, see STARD for Abstracts)	3
INTRODUCTION			
	3	Scientific and clinical background, including the intended use and clinical role of the index test	5
	4	Study objectives and hypotheses	6
METHODS			
Study design	5	Whether data collection was planned before the index test and reference standard<br>were performed (prospective study) or after (retrospective study)	6
Participants	6	Eligibility criteria	6
	7	On what basis potentially eligible participants were identified (such as symptoms, results from previous tests, inclusion in registry)	6
	8	Where and when potentially eligible participants were identified (setting, location and dates)	6
	9	Whether participants formed a consecutive, random or convenience series	6
Test methods	10a	Index test, in sufficient detail to allow replication	7
	10b	Reference standard, in sufficient detail to allow replication	7
	11	Rationale for choosing the reference standard (if alternatives exist)	
	12a	Definition of and rationale for test positivity cut-offs or result categories<br>of the index test, distinguishing pre-specified from exploratory	7
	12b	Definition of and rationale for test positivity cut-offs or result categories<br>of the reference standard, distinguishing pre-specified from exploratory	7
	13a	Whether clinical information and reference standard results were available<br>to the performers/readers of the index test	7
	13b	Whether clinical information and index test results were available<br>to the assessors of the reference standard	7
Analysis	14	Methods for estimating or comparing measures of diagnostic accuracy	8

	15	How indeterminate index test or reference standard results were handled	8
	16	How missing data on the index test and reference standard were handled	8
	17	Any analyses of variability in diagnostic accuracy, distinguishing pre-specified from exploratory	8
	18	Intended sample size and how it was determined	
RESULTS			
Participants	19	Flow of participants, using a diagram	
	20	Baseline demographic and clinical characteristics of participants	10
	21a	Distribution of severity of disease in those with the target condition	10
	21b	Distribution of alternative diagnoses in those without the target condition	10
	22	Time interval and any clinical interventions between index test and reference standard	10
Test results	23	Cross tabulation of the index test results (or their distribution) by the results of the reference standard	
	24	Estimates of diagnostic accuracy and their precision (such as 95% confidence intervals)	11-12
	25	Any adverse events from performing the index test or the reference standard	
DISCUSSION			
	26	Study limitations, including sources of potential bias, statistical uncertainty, and generalisability	15
	27	Implications for practice, including the intended use and clinical role of the index test	15
OTHER INFORMATION			
	28	Registration number and name of registry	
	29	Where the full study protocol can be accessed	
	30	Sources of funding and other support; role of funders	16

Table S1. cMyC precision profile.

Expected (pg/mL)	Mean (pg/mL)	SD	CV (%)
0	0	0.02	0
0.6	1	0.06	6
1.2	1	0.15	15
2.3	2	0.22	11
4.6	5	0.4	8
9.3	9	0.76	8.44
18.5	19	1.2	6.32
37	35	2.04	5.83
74.1	71	5.03	7.08
222.2	236	12.92	5.47
666.7	703	33.3	4.74
2000	1998	67.87	3.4

see also figure S1

Point-of-Care Testing for cMyC – preliminary results

Signal differentiation has been achieved for 10, 50 and 100 pg/mL of recombinant cMyC (C0C2 region). A combination of our antibodies 235-3H8 and 259-1A4 were used on para-magnetic and metal nano-particles (AgC and MgC) to achieve the signal (nanocoulomb) as demonstrated in figure S2.

Table S2. Baseline characteristics stratified by final diagnosis.

	**All**	**STEMI**	**NSTEMI**	**UA**	**p-value for trend**	**N**
	N=776	N=66	N=107	N=27		
Sex: male	473 (61%)	54 (82%)	75 (70%)	24 (89%)	<0.001	776
Age (years)	68 [58;78]	66 [58;75]	74 [65;81]	63 [53;68]	<0.001	776
Hypertension	439 (57%)	31 (47%)	71 (66%)	17 (63%)	0.062	776
Hyperlipidemia	622 (80%)	49 (74%)	93 (87%)	24 (89%)	0.103	776
Diabetes mellitus	147 (19%)	4 (6%)	19 (18%)	6 (22%)	0.04	776
Current smoking	230 (30%)	30 (45%)	35 (33%)	10 (37%)	0.003	776
History of smoking	217 (28%)	16 (24%)	34 (32%)	8 (30%)	0.264	776
Previous myocardial infarction	232 (30%)	11 (17%)	47 (44%)	13 (48%)	<0.001	776
Previous percutaneous intervention	200 (26%)	10 (15%)	39 (36%)	14 (52%)	<0.001	776
Systolic blood pressure (mmHg)	146 [130; 166]	141 [123; 168]	150 [132; 177]	154 [142; 169]	0.152	764
Diastolic blood pressure (mmHg)	87 [75; 99]	84 [72; 105]	91 [75; 104]	90 [84; 99]	0.208	764
Heart rate (beats/min)	84 [70; 100]	81 [62; 95]	88 [74; 102]	84 [70; 100]	0.084	765
eGFR	71 [56;86]	66 [61; 84]	70 [56; 82]	77 [66; 82]	0.455	605
Time since chest pain onset (minutes)	70 [35; 173]	71 [35; 140]	73 [39; 162]	44 [27; 125]	0.48	726

STEMI = ST elevation myocardial infarction; NSTEMI = Non-ST elevation myocardial infarction; UA = Unstable Angina; eGFR = Estimated glomerular filtration rate, ml/min/1.73m2 (estimated using the Modification of Diet in Renal Disease (MDRD) formula)

Table S3. Distribution of biomarker concentration by final adjudcated diagnostic category.

	Minimum	1st Q	Median	Mean	3rd Q	Maximum
**cMyC (ambulance, ng/L)**						
**NSTEMI**	6.6	42.4	88.0	554.1	253.1	11430
**Other**	1.9	9.1	17.4	62.8	42.7	6362
**STEMI**	7.9	48.6	306.3	1525.0	1706.0	19720
**UA**	6.8	10.7	19.4	21.6	24.8	64.72
**hs-cTnT (ambulance, ng/L)**						
**NSTEMI**	5.2	18.0	32.6	122.3	71.8	2493.9
**Other**	3.0	6.7	9.6	20.2	19.7	1035.0
**STEMI**	5.5	14.7	58.1	375.6	295.3	4023.7
**UA**	3.4	7.3	9.3	11.3	13.8	26.5

STEMI = ST-elevation Myocardial Infarction; NSTEMI = Non ST-elevation Myocardial Infarction; UA = Unstable Angina

Correlation cMyC and hs-cTnT

The biomarkers correlated positively across all patient groups (R^2=0.730, r_s=0.855) and for all patients with AMI (R^2=0.699, r_s=0.836). Table S3 and Figure S3 show the relationships between the biomarkers for each individual final adjudicated diagnosis. Serum concentrations of cMyC and hs-cTnT are positively correlated throughout, with strongest correlations observed in the non-cardiac and NSTEMI groups.

Table S4. Correlations between cMyC and hs-cTnT concentrations by diagnostic group.

Diagnosis	R^2	f	Spearman's rho	n
**NSTEMI**	0.897	913.56	0.947	107
**Other**	0.897	5000.05	0.947	576
**STEMI**	0.631	109.61	0.795	66
**UAP**	0.453	20.73	0.673	27

R^2 = correlation coefficient

Table S5. AUC values for cMyC vs hs-cTnT stratified by time since symptom onset: for early (≤60 mins), intermediate (60-120 mins), late (≥120 mins) presenters.

Subgroup	cMyC AUC	95% CI	hs-cTnT AUC	95% CI	AMI	controls	p-value*
≤60 mins	0.782	0.721-0.838	0.747	0.682-0.809	66	255	0.0528
60-120 mins	0.857	0.794-0.916	0.828	0.763-0.893	51	105	0.0917
≥120 mins	0.897	0.846-0.941	0.889	0.843-0.93	52	197	0.6349

CI = confidence interval; * p value for direct comparison AUC cMyC to hs-cTnT

Table S6. Logistic regression model statistics for derivation of figure S7.

Logistic Regression Model

		Model Likelihood Ratio Test		Discrimination Indexes		Rank Discrimination Indexes	
Obs	776	LR chi2	282.57	R2	0.467	C	0.868
0	603	d.f.	10	g	2.106	Dxy	0.736
1	173	Pr(>chi2)	<0.0001	gr	8.216	gamma	0.736
max |deriv|	2.00E-09			gp	0.256	tau-a	0.255
				Brier	0.108		
	Coef	S.E.	Wald Z	Pr(>|Z|)			
Intercept	-6.8037	1.1121	-6.12	<0.0001			
MyC_0h	1.7063	0.3436	4.97	<0.0001			
MyC_0h'	-0.7735	0.4137	-1.87	0.0615			
Creatinine	-0.0062	0.0023	-2.76	0.0057			
Sex = male	0.7497	0.2579	2.91	0.0036			
Age (y)	-0.0138	0.0094	-1.46	0.1438			
DM history = Yes	-0.9084	0.3109	-2.92	0.0035			
Chol history = Yes	0.3898	0.3082	1.26	0.2059			
HTN history = Yes	0.1877	0.2425	0.77	0.4389			
Previous MI = Yes	-0.2726	0.2446	-1.11	0.2650			
Smoking history = Yes	0.6093	0.2832	2.15	0.0314			

C = area under ROC curve, Dxy = Somers' D_{xy}, gamma = Goodman-Kruskal gamma, tau-a = Kendall's tau-a rank correlations between predicted probabilities and observed response, R^2 = Nagelkerke index, Brier score with respect to Y> its lowest level, g = Gini's mean difference (g-index), gr = g-index on the odds ratio scale, gp = g-index on the probability scale using same cut-off as used for Brier score

Table S7. Logistic regression models incorporating all variables, or cMyC and hs-cTnT alone.

Predictors used	*LR* χ^2	*Adequacy*
cMyC + clinical information	282.4	0.97
Hs-cTnT + clinical information	256.9	0.88
Combined	291.5	1.00

Table S8. Logistic regression model statistics for cMyC.

cMyC short model		Model Likelihood Ratio Test		Discrimination Indexes		Rank Discrim. Indexes	
Obs	776	LR chi2	246.49	R2	0.416	C	0.852
0	603	d.f.	5	g	1.911	Dxy	0.703
1	173	Pr(>chi2)	<0.0001	gr	6.762	gamma	0.703
max |deriv|	1e-11			gp	0.243	tau-a	0.244
				Brier	0.117		
	Coef	S.E.	Wald Z	Pr(>|Z|)			
Intercept	-7.2658	0.998	-7.28	<0.0001			
MyC_0h	1.4225	0.3136	4.54	<0.0001			
MyC_0h'	-0.6451	0.3743	-1.72	0.0848			
Sex=male	0.6316	0.2397	2.63	0.0084			
Chol history =Yes	0.2214	0.282	0.79	0.4323			
Smoking history =Yes	0.5677	0.2671	2.13	0.0336			

C = area under ROC curve, Dxy = Somers' D_{xy}, gamma = Goodman-Kruskal gamma, tau-a = Kendall's tau-a rank correlations between predicted probabilities and observed response, R^2 = Nagelkerke index, Brier score with respect to Y> its lowest level, g = Gini's mean difference (g-index), gr = g-index on the odds ratio scale, gp = g-index on the probability scale using same cut-off as used for Brier score

Using bootstrap resampling for validation, we observed modest optimism and slightly lower corrected rank discrimination indices (table S9). There were no significant interactions.

Table S9. Validation of short cMyC model used for nomogram derivation.

	index.orig	training	test	optimism	index.corrected
C	0.851	0.854	0.849	0.005	0.846
Dxy	0.703	0.708	0.698	0.011	0.692
R2	0.416	0.422	0.408	0.014	0.402
Intercept	0.000	0.000	-0.029	0.029	-0.029
Slope	1.000	1.000	0.967	0.033	0.967
Emax	0.000	0.000	0.013	0.013	0.013
D	0.316	0.322	0.309	0.013	0.304
U	-0.003	-0.003	0.000	-0.003	0.000
Q	0.319	0.324	0.309	0.015	0.304
B	0.118	0.117	0.119	-0.003	0.121
g	1.911	1.954	1.880	0.074	1.837
gp	0.2434	0.2441	0.2406	0.0035	0.2399

C = area under ROC curve, Dxy = Somers' D_{xy}, gamma = Goodman-Kruskal gamma, tau-a = Kendall's tau-a rank correlations between predicted probabilities and observed response, R^2 = Nagelkerke index, Brier score with respect to Y> its lowest level, g = Gini's mean difference (g-index), gr = g-index on the odds ratio scale, gp = g-index on the probability scale using same cut-off as used for Brier score

Table S10. Discriminatory power of cMyC at different thresholds.

**cMyC Diagnostic proportions - all patients**			
**[cMyC]**	10 ng/L	87 ng/L	120 ng/L
**Sensitivity**	96.6% (93.5-98.9%)	54.7% (47.6-62.1%)	46.1% (38.7-53.7%)
**Specificity**	29.2% (25.5-33%)	90.2% (87.6-92.6%)	92.2% (90-94.3%)
**NPV**	96.8% (93.8-99%)	87.4% (84.7-90%)	85.6% (82.7-88.2%)
**PPV**	28.1% (24.5-31.8%)	61.4% (54-69.6%)	62.7% (54.6-71.3%)

Diagnostic proportions of cMyC and hs-cTnT. NPV = Negative Predictive Value; PPV = Positive Predictive Value

Table S11. Discriminatory power of hs-cTnT at different thresholds – stratified by time since chest pain onset.

**Patients with chest pain for <60 mins**				
**[hs-cTnT]**	5 ng/L	14 ng/L	50 ng/L	52 ng/L
**Sensitivity**	100% (100-100%)	66.7% (54.8-77%)	27.3% (17.4-38.2%)	27.3% (17.4-38.2%)
**Specificity**	13.8% (9.7-18%)	67.1% (61.1-72.7%)	94.2% (91-96.9%)	94.2% (91-96.9%)
**NPV**	100% (100-100%)	88.8% (83.7-92.7%)	83.6% (79.2-87.6%)	83.6% (79.2-87.6%)
**PPV**	22.9% (17.9-28%)	34.1% (26-42.7%)	54.8% (36.7-71.9%)	54.8% (36.7-71.9%)
**Patients with chest pain for 60-120 mins**				
**Sensitivity**	100% (100-100%)	84.5% (72.7-93.8%)	39.3% (25.5-52.8%)	39.3% (25.5-52.8%)
**Specificity**	7.5% (2.9-13.5%)	65.1% (55.8-73.8%)	93.6% (88.2-98.1%)	94.6% (89.6-98.9%)
**NPV**	100% (100-100%)	89.8% (82.1-95.9%)	76% (68.9-82.8%)	76.2% (69-83%)
**PPV**	34.5% (27-42.1%)	53.8% (43-65.3%)	75% (57.7-91.7%)	78.2% (60.7-93.3%)
**Patients with chest pain for ≥120 mins**				
**Sensitivity**	100% (100-100%)	94.3% (86-100%)	57.9% (43.5-71.1%)	56% (41.8-68.8%)
**Specificity**	15.8% (11.2-20.8%)	62.3% (55.7-69.6%)	92.5% (88.7-95.8%)	93% (89.2-96.2%)
**NPV**	100% (100-100%)	97.7% (94.6-100%)	89.3% (84.7-93.3%)	89% (84.4-92.8%)
**PPV**	23.6% (18.3-29.6%)	39.7% (30.9-48.4%)	66.7% (52.4-80%)	67.4% (52.5-81.6%)

NPV = Negative Predictive Value; PPV = Positive Predictive Value

Table S12. Discriminatory power of hs-cTnT at different thresholds – for all patients.

hs-cTnT Diagnostic proportions - all patients				
**[hs-cTnT]**	5 ng/L	14 ng/L	50 ng/L	52 ng/L
**Sensitivity**	100% (100-100%)	80.5% (73.8-86.3%)	40.4% (33.3-48.4%)	40% (32.7-47.6%)
**Specificity**	13.4% (10.8-16.2%)	65.1% (61.4-68.9%)	93.5% (91.2-95.3%)	93.8% (91.7-95.6%)
**NPV**	100% (100-100%)	92.1% (89.4-94.6%)	84.6% (81.8-87.5%)	84.5% (81.5-87.4%)
**PPV**	24.7% (21.5-28.2%)	39.7% (34.3-45%)	63.9% (54.6-72.7%)	64.7% (55.4-74%)

NPV = Negative Predictive Value; PPV = Positive Predictive Value

Table S13. Reclassification analysis for cMyC vs hs-cTnT.

hs-cTnT	cMyC							
	Non-AMI				AMI			
	Rule-Out	Observe	Rule-In	Reclassified	Rule-Out	Observe	Rule-In	Reclassified
Rule-Out	20	4	0	17%	0	0	0	0%
Observe	39	485	17	10%	0	91	13	12%
Rule-In	0	8	30	21%	0	2	67	3%
NRI categorical	0.1067 (95% CI, 0.0563-0.1571); p <0.001							
IDI	0.032 (95% CI, 0.0168-0.0472); p <0.001							

Reclassification analysis for cMyC and hs-cTnT in a Net Reclassification Table; based on sensitivity & NPV of cMyC in the cohort, patients were eligible for rule-out with chest pain >120 mins and cMyC <10 ng/L at first blood draw; rule-in if cMyC ≥120 ng/L. For hs-cTnT, the triage was modelled on a first blood draw as per ESC 0/1h-algorithm – direct rule-out if chest pain ≥ 180 mins and hs-cTnT < 5 ng/L; rule-in if hs-cTnT ≥52 ng/L. NRI = Net Reclassification Benefit; IDI = Integrated Discrimination Improvement

Table S14. Prediction of death and first non-fatal MI/death during follow-up.

Model i) Death during FU

		Model Tests		Discrimination Indexes	
Obs	769	LR chi2	110.83	R2	0.179
Events	81	d.f.	5	C	0.798
Center	6.94	Pr(>chi2)	0	Dxy	0.597
		Score chi2	123.33	g	1.71
		Pr(>chi2)	0	gr	5.527
	Coef	S.E.	Wald Z	Pr(>|Z|)	
MyC_0h	0.892	0.417	2.140	0.032	
MyC_0h'	-0.644	0.446	-1.450	0.148	
creatinine	0.002	0.001	2.540	0.011	
age_y	0.058	0.012	4.930	<0.0001	
previousMI=Yes	0.582	0.226	2.570	0.010	

Model ii) Non-fatal AMI or Death during FU

		Model Tests		Discrimination Indexes	
Obs	771	LR chi2	285.66	R2	0.317
Events	228	d.f.	3	C	0.828
Center	4.8164	Pr(>chi2)	0	Dxy	0.656
		Score chi2	405.45	g	1.558
		Pr(>chi2)	0	gr	4.75
	Coef	S.E.	Wald Z	Pr(>|Z|)	
MyC_0h	1.633	0.224	7.300	<0.0001	
MyC_0h'	-1.173	0.239	-4.900	<0.0001	
dm_base=Yes	-0.407	0.174	-2.340	0.019	

Model statistics to predict probability of i) death and ii) non-fatal AMI or death during follow-up

Table S15. Cox regression model for outcome death.

Variable	*HR*	*95% CI*	*p-value*
[creatinine]	1.002	1.001-1.004	0.003
previous MI = Yes	1.794	1.120-2.872	0.015
log [cMyC]	1.355	1.193-1.54	<0.001
Age (years)	1.07	1.045-1.095	<0.001
Likelihood ratio test	101.6 on 4 degrees of freedom, p <0.001		
Wald test	89.17 on 4 degrees of freedom, p <0.001		

Cox regression model for outcome death during 2-year follow-up, for variables used in the nomogram creation; HR = hazard ratio; CI = confidence interval

Figure S1. cMyC assay precision profile.

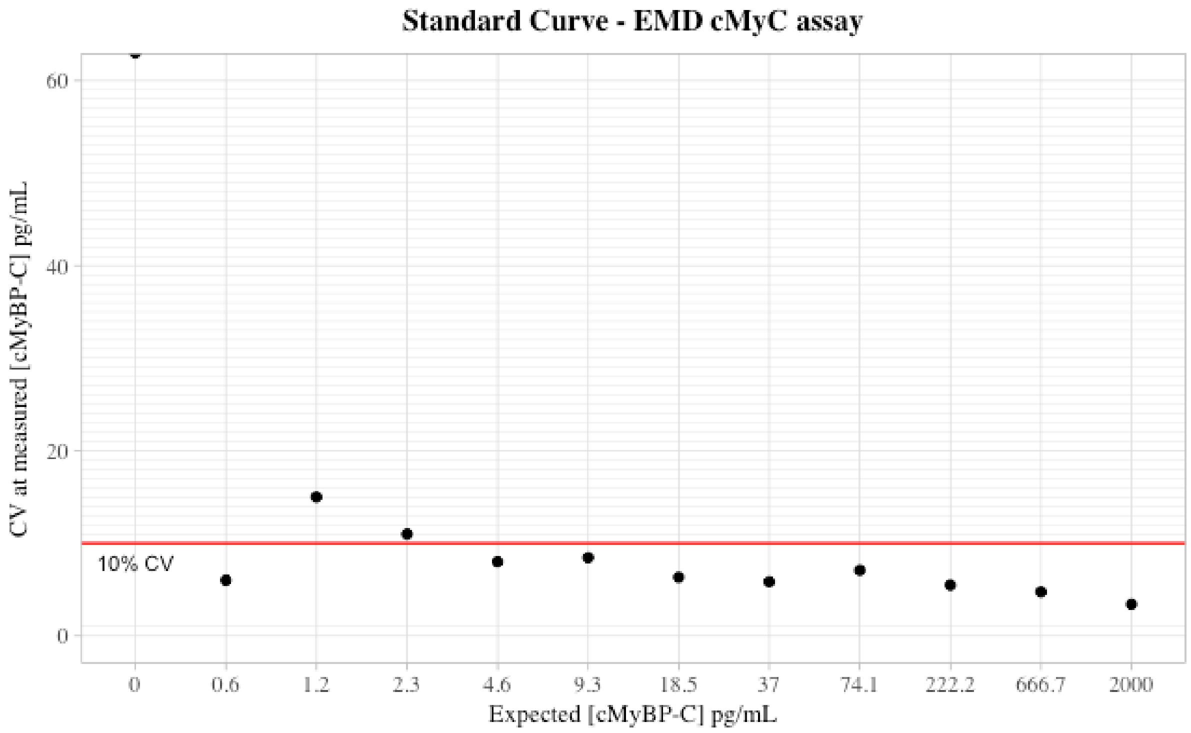

Figure S2. Signal obtained for AgC (235-3H8) against MgC (259-1A4) for varying concentrations of C0C2 analyte.

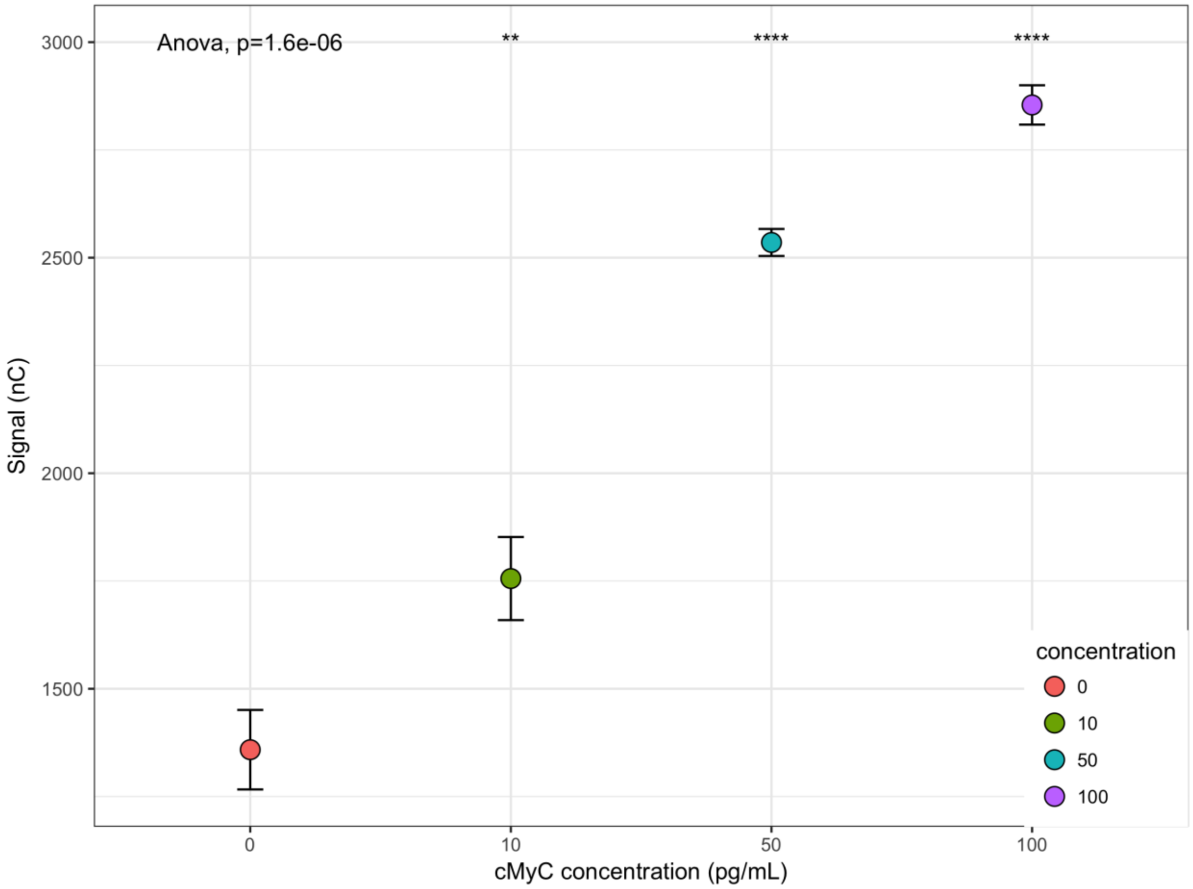

Points represent mean concentration, error bars the standard error of the mean. Significance tests have been performed comparing all groups (Anova, as printed) and as unpaired T-test against concentration 0: **: $p \leq 0.01$; ****: $p \leq 0.0001$; CV: 10% at 10 pg/mL; 2% at 50 pg/mL, 3% at 100 pg/mL

Figure S3. Scatter plots outlining correlation between cMyC and hs-cTnT concentrations (ng/L both) in samples obtained in the ambulance for each diagnostic group.

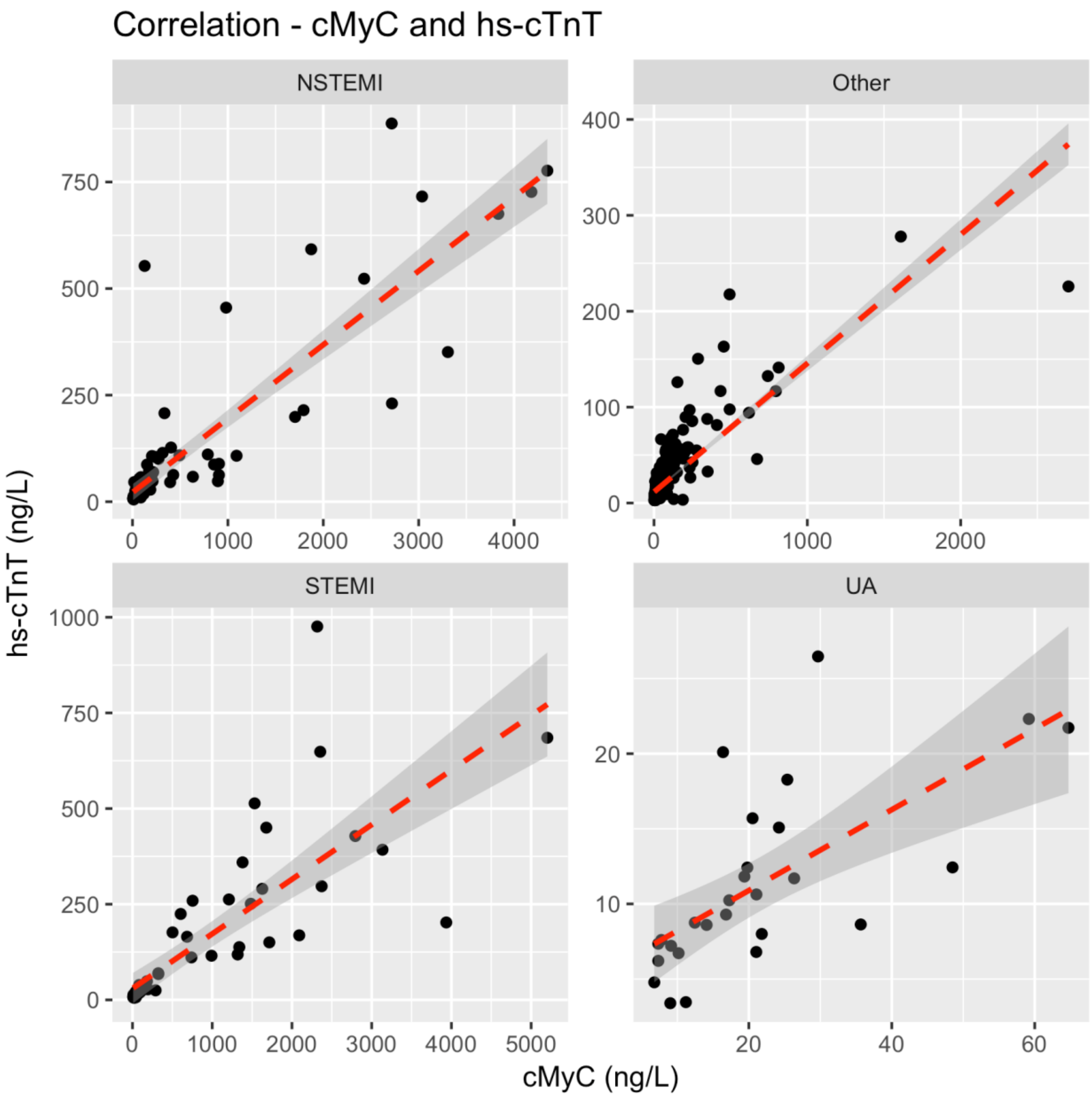

Light grey shading depicts the boundaries of the 95% confidence intervals, line of best fit indicated in red. NSTEMI = Non-ST elevation Myocardial Infarction; STEMI = ST-elevation Myocardial Infarction; UA = Unstable Angina

Figure S4. Histogram for hs-cTnT concentrations from pre-hospital samples, stratified by diagnosis of AMI; x-axis log10-transformed.

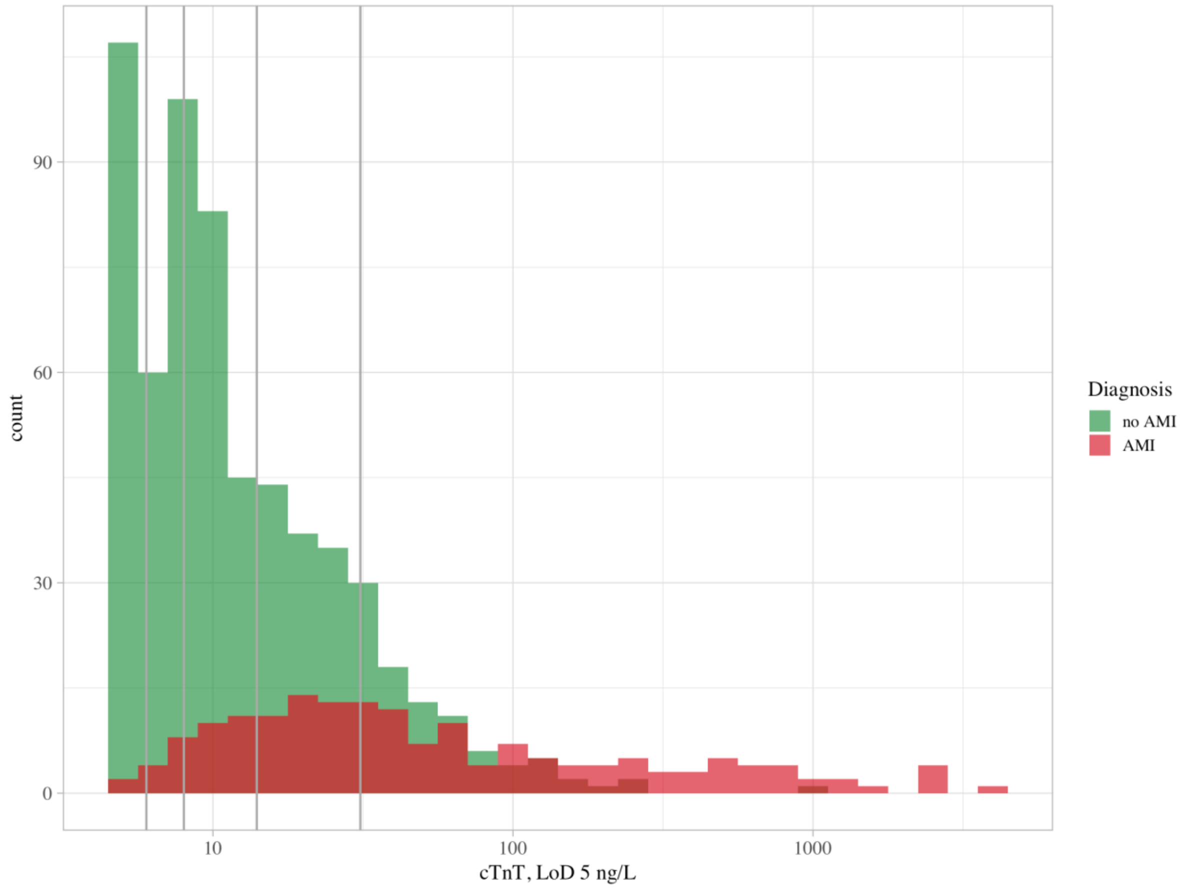

Figure S5. Receiver-operating characteristics (ROC) curves for cMyC (ambulance) and hs-cTnT (ambulance) for the diagnosis of NSTEMI.

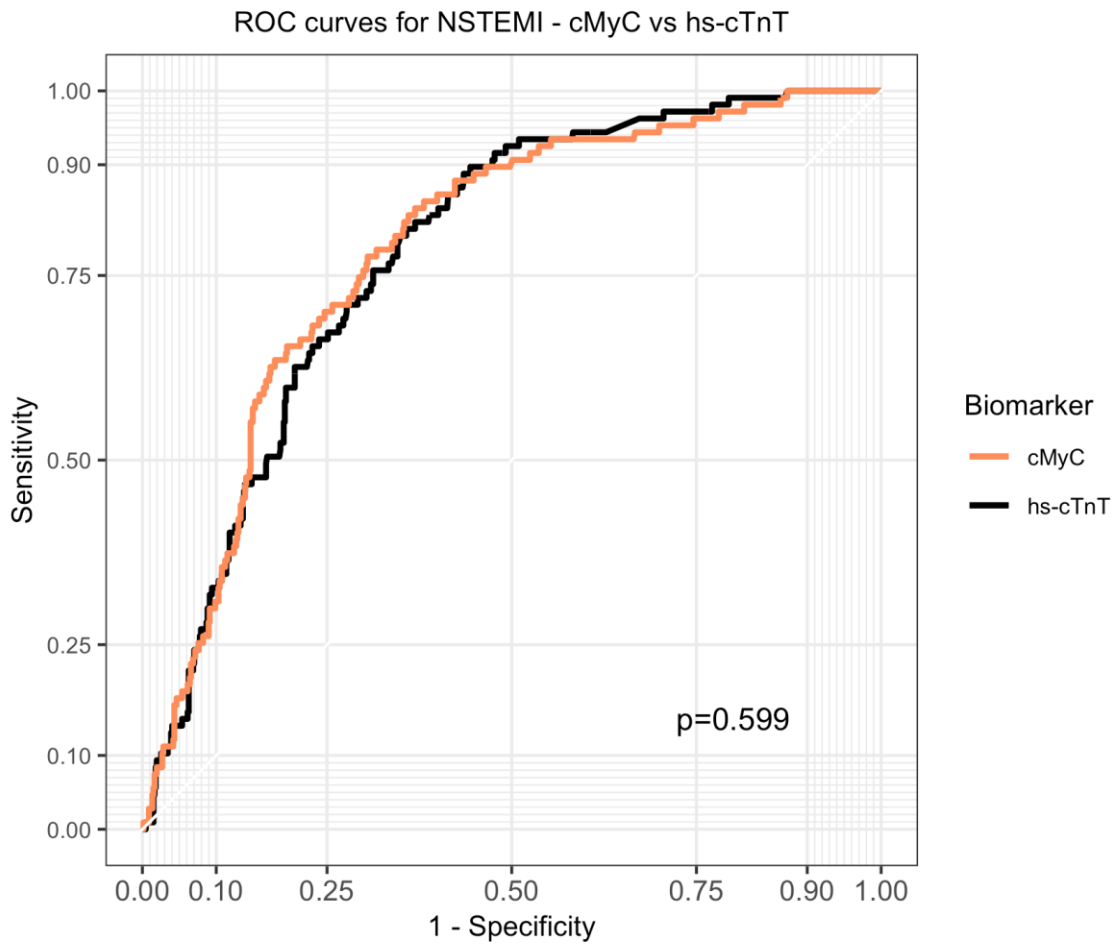

The AUC for cMyC 0.787 (95% CI, 0.741-0.829), for hs-cTnT 0.781 (95% CI, 0.737-0.820; p=0.595 for direct comparison to cMyC).

Figure S6. Receiver-operating characteristics (ROC) curves for cMyC (ambulance) and hs-cTnT (ambulance) for the diagnosis of STEMI.

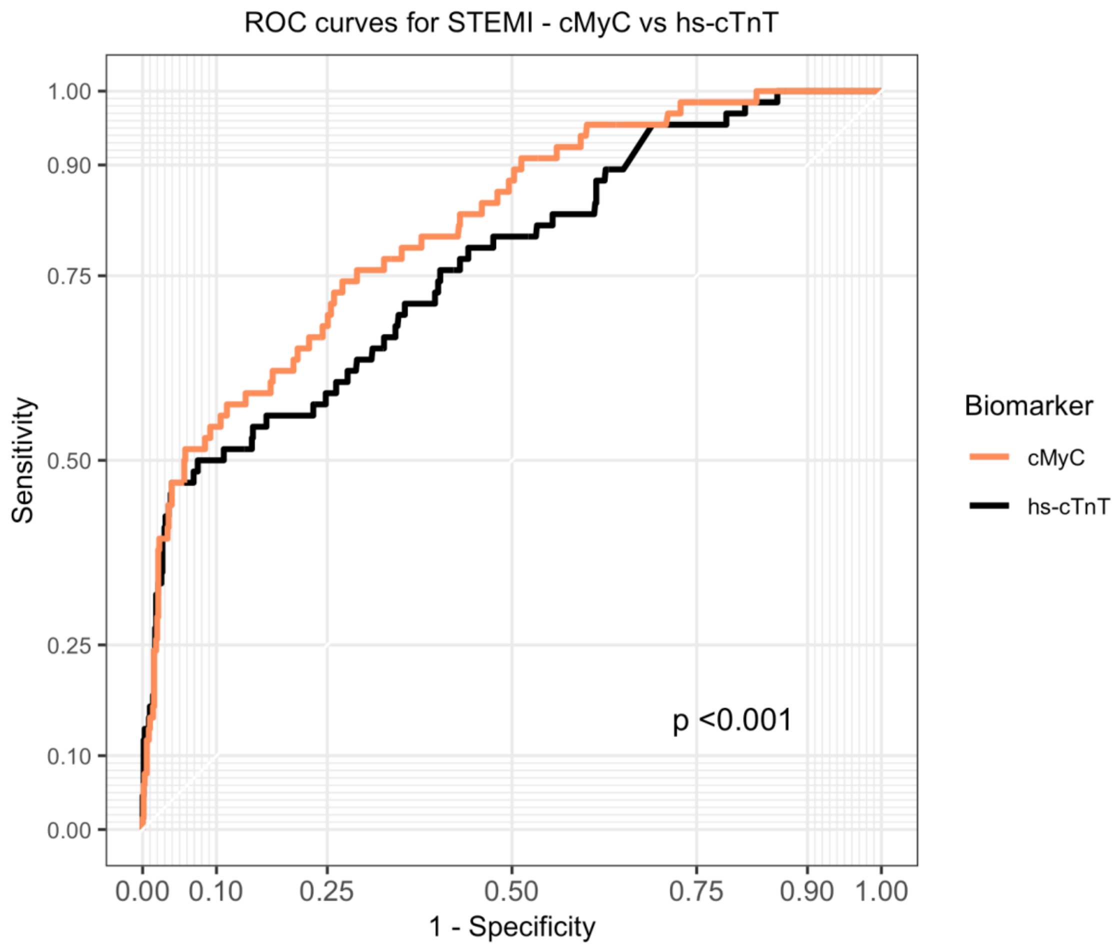

The AUC for cMyC was 0.816 (95% CI, 0.761-0.866), for hs-cTnT 0.766 (95% CI, 0.701-0.828; p<0.001 for direct comparison to cMyC).

Figure S7. Odds ratio for AMI diagnosis at presentation based on [cMyC] and stratified by [creatinine]; facetted by sex (horizontal), and history of diabetes mellitus (vertical); other variables held stable.

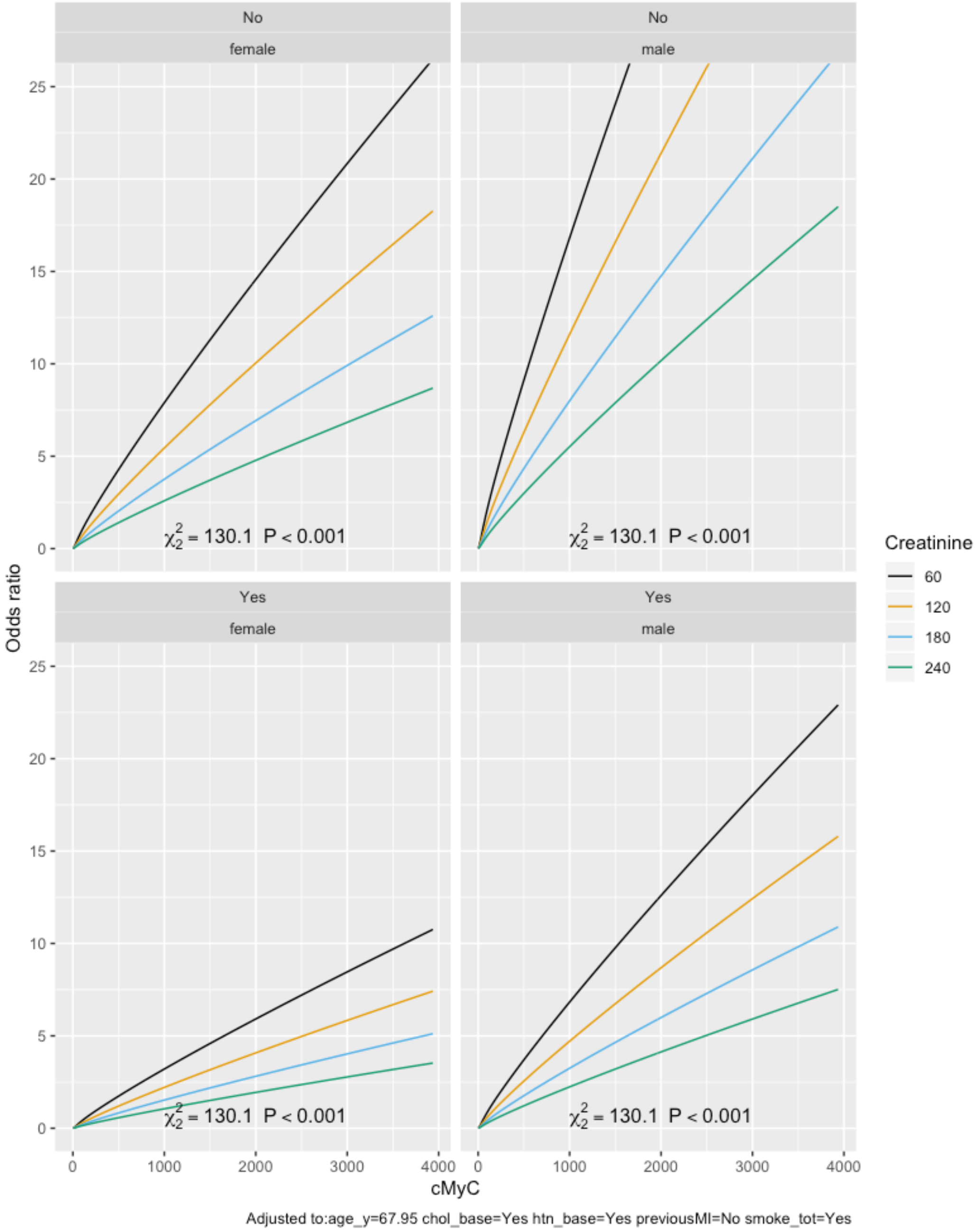

The model was derived using a multivariable logistic regression model as outlined in table S5.

Figure S8. Calibration plot for complete model, validated using 150 bootstrap repetitions.

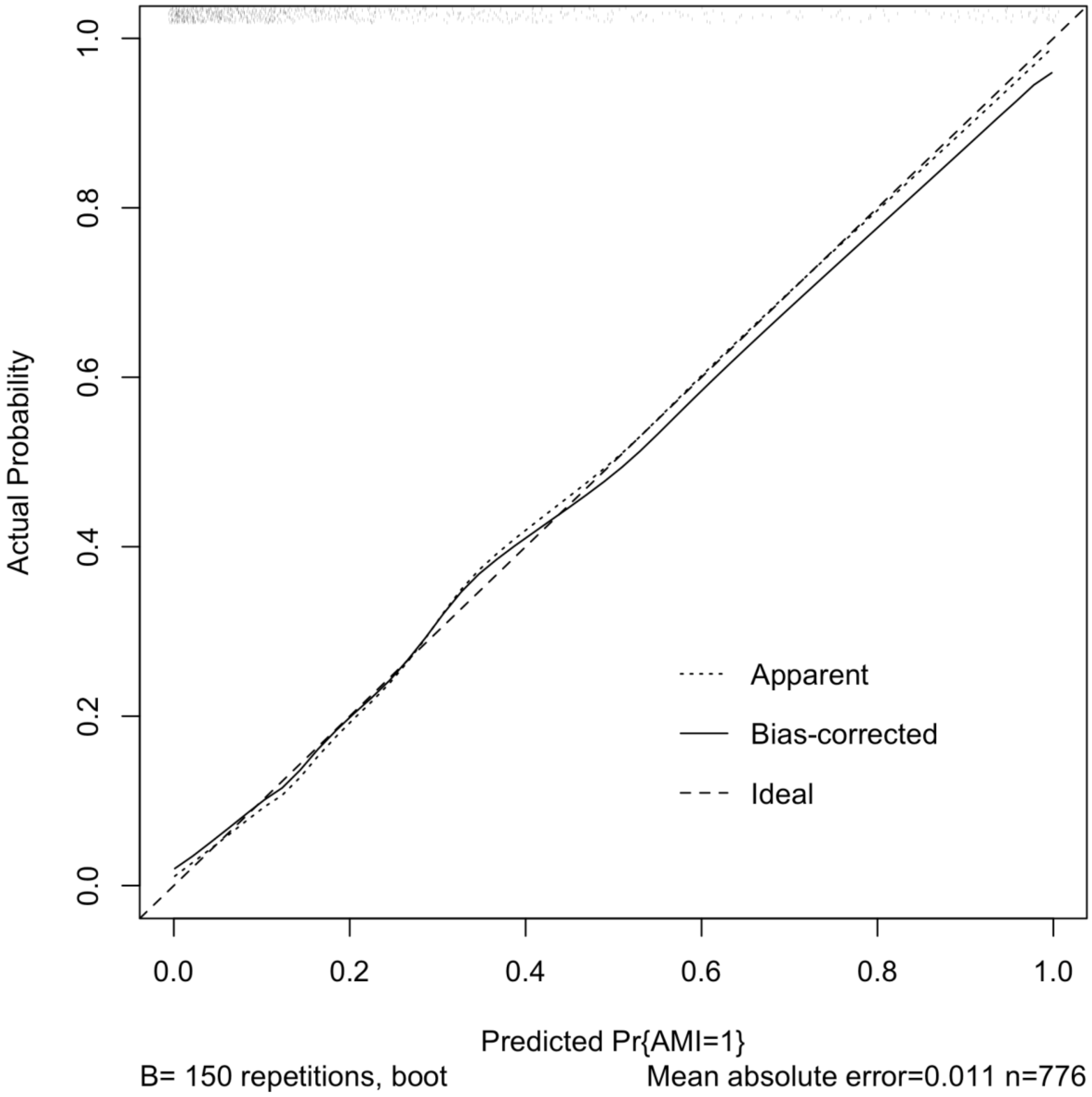

A nonparametric calibration curve is estimated over a sequence of predicted values.

Figure S9. Nomogram for the use of cMyC concentration, creatinine concentration, age and history of prior myocardial infarction to predict probability of death during follow-up.

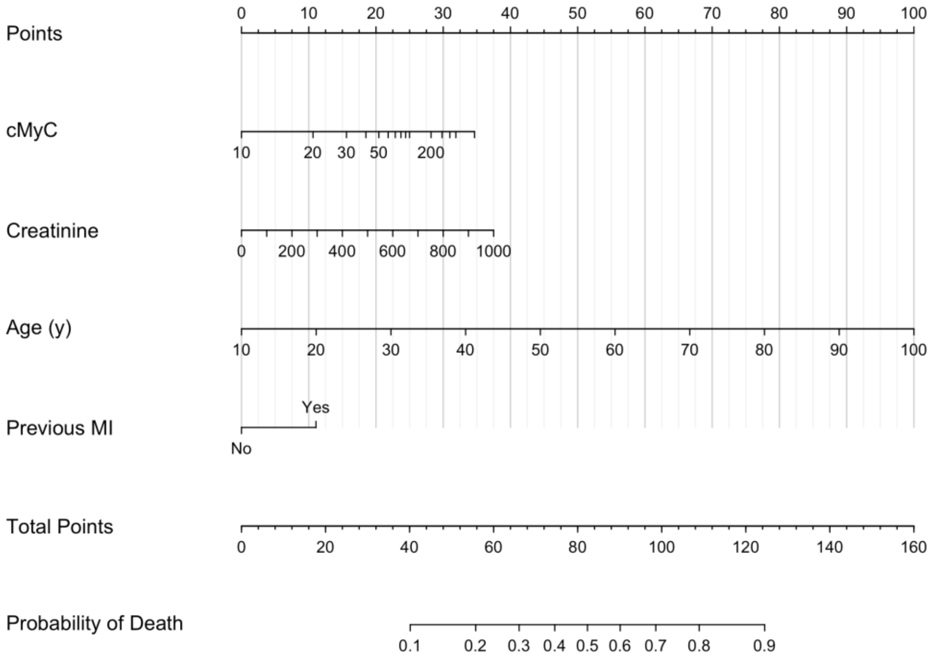

Each variable scores on the points scale on top of the nomogram, and the respective values are added up to the complete score – the total point scale then allows to transfer the sum of all predictors to scale for the 'probability of death' during 2-year follow-up.

Figure S10. Nomogram for the use of cMyC concentration and history of diabetes mellitus to predict probability of non-fatal MI or death during follow-up.

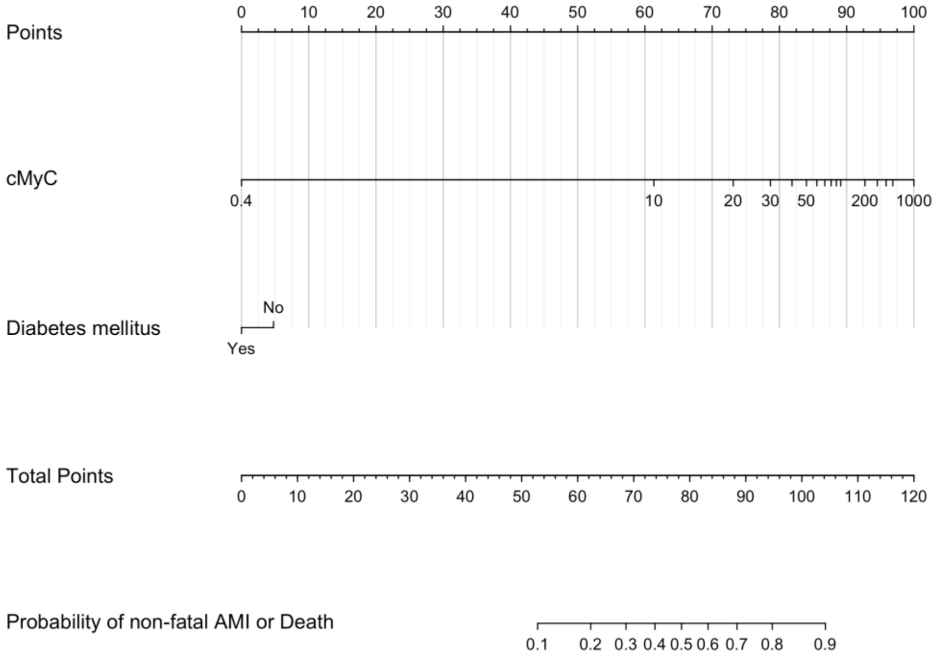

Each variable scores on the points scale on top of the nomogram, and the respective values are added up to the complete score – the total point scale then allows to transfer the sum of all predictors to scale for the 'probability of death' during 2-year follow-up.

Figure S11. Facet plots describing effect of increasing cMyC concentration and prior myocardial infarction on the probability of death during follow-up.

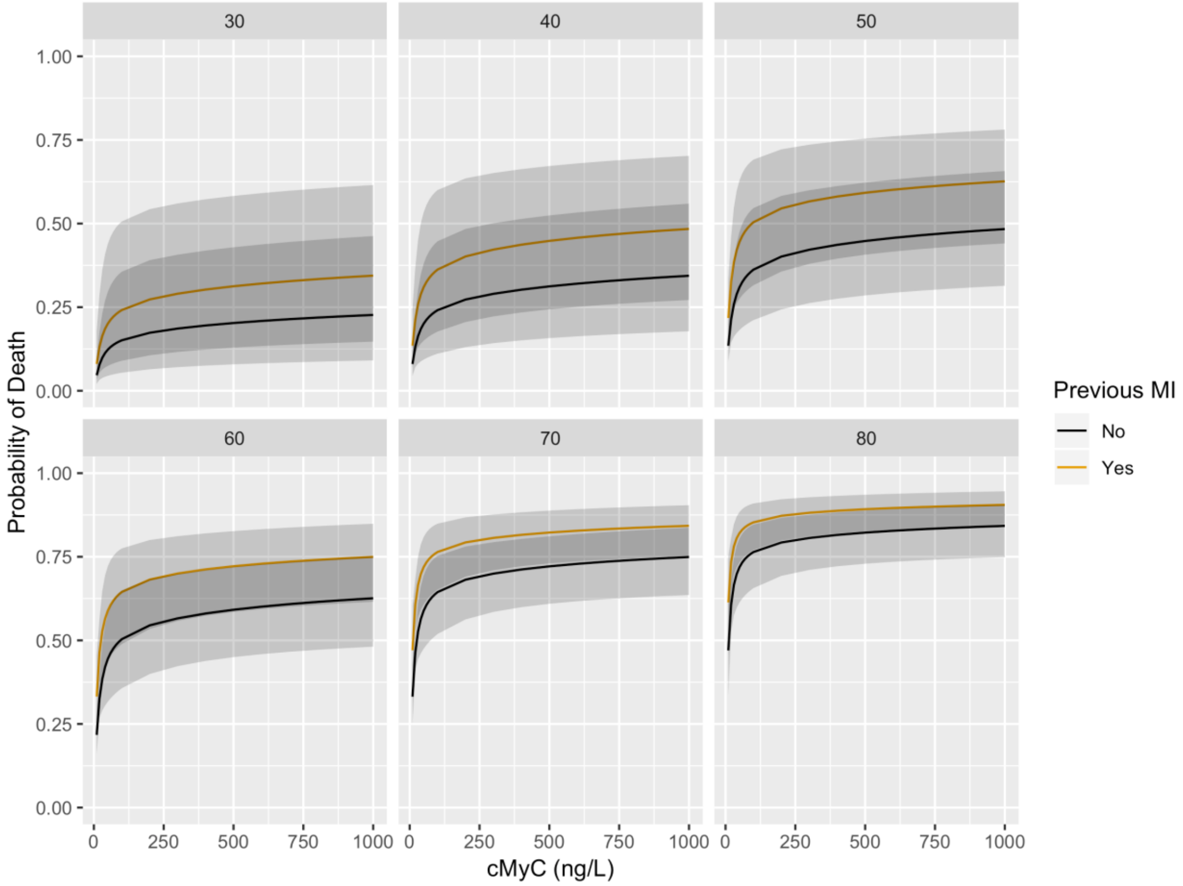

Facets represent age categories.

Figure S12. Cumulative event (mortality) curves for all patients over a 2-year follow-up for cMyC from samples obtained in the ambulance.

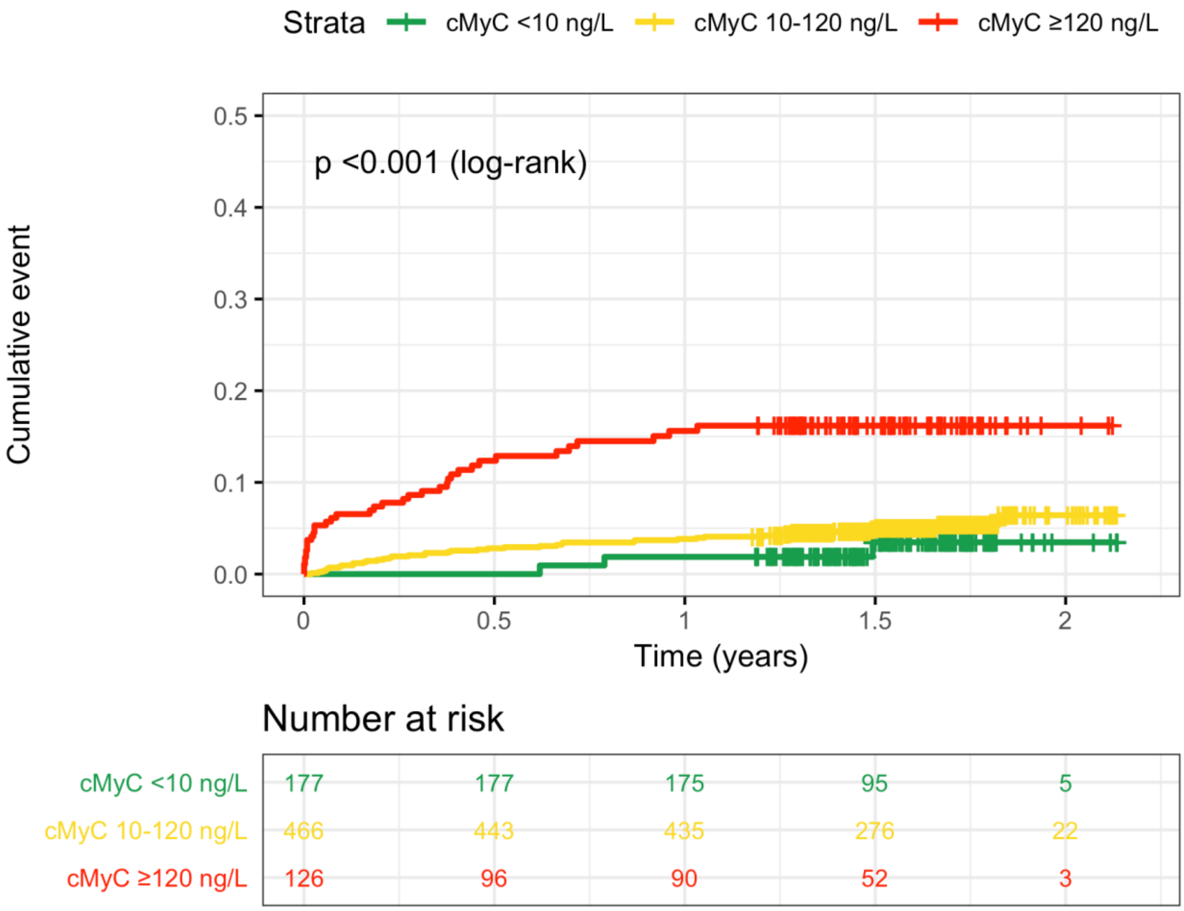

These are adjusted for the Cox model (using age (in years), presence of baseline diabetes mellitus and prior myocardial infarction as significant covariates) and stratified for the following cMyC levels: <10 ng/L, 10-120 ngL, ≥120 ng/L.
